# Systematic immune cell dysregulation and molecular subtypes revealed by single-cell RNA-seq of subjects with type 1 diabetes

**DOI:** 10.1186/s13073-024-01300-z

**Published:** 2024-03-27

**Authors:** Mohammad Amin Honardoost, Andreas Adinatha, Florian Schmidt, Bobby Ranjan, Maryam Ghaeidamini, Nirmala Arul Rayan, Michelle Gek Liang Lim, Ignasius Joanito, Quy Xiao Xuan Lin, Deepa Rajagopalan, Shi Qi Mok, You Yi Hwang, Anis Larbi, Chiea Chuen Khor, Roger Foo, Bernhard Otto Boehm, Shyam Prabhakar

**Affiliations:** 1 Laboratory of Systems Biology and Data Analytics, Genome Institute of Singapore (GIS), A*STAR (Agency for Science, Technology and Research), Singapore, 138672 Singapore; 2 Cardiovascular Diseases Translational Research Program, Yong Loo Lin School of Medicine, National University of Singapore, Singapore, 117599 Singapore; 3 School of Public Health and Preventive Medicine, Monash University, Melbourne, Australia; 4 Integrated genomics platform, Genome Institute of Singapore (GIS), A*STAR (Agency for Science, Technology and Research), Singapore, 138672 Singapore; 5 Singapore Immunology Network (SIgN), A*STAR (Agency for Science, Technology and Research), Singapore, 138648 Singapore; 6 Institute of Molecular and Cell Biology (IMCB), A*STAR (Agency for Science, Technology and Research), Singapore, 138673 Singapore; 7 Genome Institute of Singapore (GIS), A*STAR (Agency for Science, Technology and Research), Singapore, 138672 Singapore; 8 Lee Kong Chian School of Medicine, Nanyang Technological University, Singapore, 308232 Singapore; 9 Faculty of Life Sciences and Medicine, King’s College London, London, WC2R 2LS UK; 10 Cancer Science Institute of Singapore, National University of Singapore, Singapore, Republic of Singapore

**Keywords:** Type 1 diabetes mellitus (T1DM), Peripheral blood mononuclear cells (PBMCs), Single-cell RNA sequencing (scRNA-seq)

## Abstract

**Background:**

Type 1 diabetes mellitus (T1DM) is a prototypic endocrine autoimmune disease resulting from an immune-mediated destruction of pancreatic insulin-secreting $\beta$ cells. A comprehensive immune cell phenotype evaluation in T1DM has not been performed thus far at the single-cell level.

**Methods:**

In this cross-sectional analysis, we generated a single-cell transcriptomic dataset of peripheral blood mononuclear cells (PBMCs) from 46 manifest T1DM (stage 3) cases and 31 matched controls.

**Results:**

We surprisingly detected profound alterations in circulatory immune cells (1784 dysregulated genes in 13 immune cell types), far exceeding the count in the comparator systemic autoimmune disease SLE. Genes upregulated in T1DM were involved in WNT signaling, interferon signaling and migration of T/NK cells, antigen presentation by B cells, and monocyte activation. A significant fraction of these differentially expressed genes were also altered in T1DM pancreatic islets. We used the single-cell data to construct a T1DM metagene z-score (TMZ score) that distinguished cases and controls and classified patients into molecular subtypes. This score correlated with known prognostic immune markers of T1DM, as well as with drug response in clinical trials.

**Conclusions:**

Our study reveals a surprisingly strong systemic dimension at the level of immune cell network in T1DM, defines disease-relevant molecular subtypes, and has the potential to guide non-invasive test development and patient stratification.

**Supplementary Information:**

The online version contains supplementary material available at 10.1186/s13073-024-01300-z.

## Background

Type 1 diabetes mellitus (T1DM) is a prototypic endocrine autoimmune disease driven by chronic inflammatory responses against insulin-secreting pancreatic $\beta$ cells, leading to insulin deficiency with life-long dependence on exogenous insulin treatment [1]. The disease is characterized by chronic inflammation with impaired homeostasis at the level of innate and adaptive immune cells. The existing therapies for T1DM are still limited in terms of long-term efficacy and addressing the underlying pathophysiology [2, 3]. Patient heterogeneity has been considered a key factor in treatment response [2]. Moreover, data suggest that diverse immune cell types and signaling pathways contribute to T1DM pathology [4]. Thus, a comprehensive analysis of immune cell phenotypes, cell type-specific molecular traits and their potential variation across affected individuals is called for [5].

A related challenge is that T1DM-related molecular mechanisms are poorly understood in humans. Mechanistic studies are hindered by the fact that pancreatic islets, which are the primary target of autoimmune attack, are difficult to access in living individuals. However, multiple lines of evidence indicate that systemic immune cell dysregulation, including Treg and NK cells, precedes T1DM diagnosis, and is implicated in disease pathogenesis and progression [6–12]. Furthermore, fine-mapping of genetic risk factors indicates that T1DM-associated risk variants exert a significant impact on the transcriptomes of circulating immune cells [13–16]. Consequently, non-invasive approaches using peripheral blood samples have become an essential strategy for identification of markers related to the progression of the autoimmune disease.

Along this line, immunological studies of T1DM have greatly advanced the field, for example by highlighting the role of specific, disease-predisposing human leukocyte antigen (HLA) haplotypes [17, 18], by revealing insulin, GAD-65, and IA-2A as well as $\beta$ cells proteins as common autoantigens [19–21], and by identifying common aberrations in cell composition and gene expression in peripheral immune cells [6, 7, 22–25]. Of note, most studies have examined the pre-diabetic molecular immune signature of first-degree relatives (FDR) of T1DM subjects [26]. In contrast to T1DM cases in the general population (GP), FDR T1DM subjects exhibit a lower age at diagnosis [27] and are enriched for monogenic variants of significant impact [28]. Moreover, the vast majority of existing molecular analyses are based on analysis of bulk samples, which obscures the heterogeneity of immune cell types and cell states. Consequently, our understanding of molecular and cellular abnormalities in GP T1DM is still limited, as is our ability to identify patient subtypes.

Recently, single-cell RNA-seq (scRNA-seq) profiling of ex vivo (cultured) human pancreatic islets shed light on cellular-resolution gene expression signatures in T1DM [29]. However, since immune cells comprised only a minor fraction of cells identified, this study focused primarily on endocrine and exocrine cell types. Another study of T1DM-related immune cell phenotypes analyzed scRNA-seq data from PBMCs of 4 T1DM subjects and 4 controls and identified *IL32*-expressing cells as a signature of T1DM [24]. In view of the current situation, there is an unmet need for a more comprehensive analysis of immune cells in T1DM.

We hypothesized that T1DM, an organ specific autoimmune disease, may involve systemic immune perturbation in cell type abundance as well as cell type-specific gene expression. In this cross-sectional study, we used the technique of scRNA-seq to characterize PBMCs from 46 islet-cell autoantibody-positive manifest T1DM cases (stage 3) and 31 healthy controls matched for age, gender, and presence of T1DM-associated risk HLA haplotypes. Our analysis revealed shifts in cellular composition as well as profound molecular aberrations in 12/14 peripheral immune cell types from T1DM subjects. Notably, we identified widespread upregulation of genes associated with immune cell activation, though regulon analysis indicated that this process may be driven by distinct transcription factors (TFs) in T, B, and myeloid cells. The upregulated and downregulated genes we identified in PBMC cell types showed significant overlap with transcriptome changes in T1DM pancreas, suggesting molecular links between systemic and organ-specific immune responses [18, 29]. Our differentially expressed genes (DEGs) also showed significant overlap with markers that predict seroconversion (appearance of autoantibodies in peripheral blood) or T1DM onset in high-risk individuals. We also used the single-cell data to define cell type-specific metagene expression scores segregating cases and controls. Importantly, these metagene scores were highly correlated across cell types, even though the DEG sets were largely non-overlapping. To characterize immune variation across the cohort, we therefore aggregated scores across cell types to define a single composite score for each sample, which we defined as the T1DM metagene *Z*-score (TMZ score). The TMZ score for each individual correlated with GAD autoantibody (GADA) titer and the number of high-risk HLA haplotypes as well as with drug response in interventional clinical trials. Finally, we found that DEGs in effector T cells and B cells were enriched for T1DM genetic risk.

In summary, our case-control study shows unexpectedly strong systemic immune cell dysregulation in T1DM, defines disease-relevant T1DM molecular subtypes, and has the potential for non-invasive test development in T1DM.

## Methods

### Sample collection and cohort details

European stage 3 T1DM subjects were selected from a cohort of 1634 T1D patients from Baden-Wuerttemberg in Germany. The healthy controls matched for age, gender, and T1DM risk HLA haplotypes were selected from a population-based cohort of more than 8367 subjects, again from Baden-Wuerttemberg. To rule out the presence of an infection or any other inflammatory process, we selected subjects with CRP below 1 mg/L (high sensitivity C-reactive protein (hs-CRP); Roche Cobas 6000 analytical system). BMI was restricted within the range from 18.5 to 24.9 kg/m^2^, i.e., the WHO recommendation of a normal BMI for Europeans. Additional inclusion criteria were as follows: normal aspartate transaminase (AST) levels (below 33 units per liter (U/L) and alanine transaminase (ALT) levels (below 40 units per liter (U/L), normal creatine serum values (below 110 µmol/L for men, below 95 µmol/L for women) resulting in normal values of the estimated glomerular filtration rate (eGFR), normal TSH level (range 0.3–4 mU/l) indicative of normal thyroid function, TPO antibody negative, normal vitamin B_12_ serum levels (range between 350 and 900 pg/ml), normal 25-OH Vit D levels (range between 30 and 70 µg/l), normal levels of plasma ferritin (ranges between 50 and 330 ng/mL) as a marker of a normal iron status as well as a surrogate marker of inflammation, and normal serum zinc levels (range between 70 and 120 µg/dL) to show an adequate nutritional status and to rule out problems absorbing nutrients.

All control subjects were negative for ICA, GADA, IA-2A, and ZnT8 antibodies. The T1D subjects were positive for at least 2 islet cell autoantibodies. All T1D subjects selected were GADA positive [30, 31]. The Roche Elecsys C-Peptide assay was used to quantify fasting C-peptide levels in T1D subjects. All T1D subjects revealed fasting C-peptide levels below 0.01 ng/mL and were therefore considered as “C-peptide negative.” In addition, all T1D subjects were requiring insulin doses above 0.65 units of insulin per kg body weight per day. None of the T1D subjects had evidence of microalbuminuria as a marker of diabetic nephropathy or evidence of diabetic retinopathy. In subjects with T1DM, blood glucose during the blood collection had to be within the range of a predefined target, i.e., 80–140 mg/dl to exclude a potential impact of elevated glucose levels, altered plasma cortisol and catecholamine levels on immune cell counts. Also, HbA1c levels had to label a well-controlled diabetes status with a level below 7.5% (DCCT/NGSP; corresponding to a level below 58 mmol/mol (IFCC; SI unit) [32–34]. Since the counts of lymphocytes as well as lymphocyte subsets are known to be influenced by hormonal factors and temperature, to achieve pre-analytical consistency, the blood collection was only performed in the morning (8:00–10:00 am) at a fasting state during the seasonal period of spring until autumn. All technical procedures and protocols for the recruitment, blood collection, and PBMC isolation were reviewed and approved by the Institutional Review Board (IRB) at the Division of Endocrinology and Diabetes in Ulm University, Germany (IRB no. 299604393*R*) [35]. Accordingly, PBMCs were isolated from approximately 10 ml of heparinised whole blood using standard Ficoll-Hypaque density gradient centrifugation method. Isolated PBMCs were frozen in Fetal bovine serum (FBS) containing 10% dimethylsulfoxide (DMSO) and shipped to Genome Institute of Singapore for downstream experiments. The sequence-specific oligonucleotide DNA amplification method was utilized for HLA class II genotyping using *HLA-DRB1*, *HLA-DQA1*, and *HLADQB1* specific primers [36, 37]. Autoantibody assay for detection of two main autoantibodies in T1DM (i.e., GADA and IA-2A) was conducted based on previously established fluid-phase-antigen-binding assay [38]. The clinical information of all samples included in current study are summarized in the Additional file 2: Table S1-S2.

### Sample preparation and scRNA-seq

Frozen PBMCs samples were thawed in 10 ml of pre-warmed thawing media (RPMI 1640 supplemented with 5% human serum, 1% penicillin-streptomycin, and 1% glutamine). After centrifugation at 300g, the supernatant was removed and cells were resuspended in 10 ml of washing media (RPMI 1640 supplemented with 10% fetal bovine serum (FBS), 1% penicillin-streptomycin, 1% glutamine). Cells were washed two times with phosphate-buffered saline (PBS) containing 0.04% bovine serum albumin (BSA) and filtered through a 5-µm cell strainer. The cell concentration and viability were measured in trypan blue using the Bio Rad TC20^TM^ Automated Cell Counter (Bio-Rad). Droplet-based scRNA-seq was applied to the cohort samples using the single-cell 3′ reagent kit (V2, 10x Genomics), according to the manufacturer’s protocol. Single-cell suspensions from four samples (two cases and two healthy controls) were mixed at the final concentration of $1 \times 10^{6}$ cells/ml. Before mixing samples, an aliquot of 2 million cells from each sample was centrifuged and kept at − 80^o^ C and used for DNA extraction and genotyping. Pooled cell suspensions were loaded onto the ChromiumTM Controller instrument at the recommended volume required for capturing 16,000 cells per pool. The cDNA amplification and library preparation were performed using the library construction kit (10x Genomics). Quality, size distribution, and quantity of generated cDNA and sequencing libraries were confirmed using the High Sensitivity DNA kit (Agilent) and Kapa kit (Illumina). After quality control, pooled libraries were diluted in final concentration of 10 nM and were sequenced on Illumina HiSeq 4000 to get on average read-depth of 80,000 reads/cell using following chemistry settings: read 1: 26 cycles; i7 index: 8 cycles; i5 index: 0 cycles; and read 2: 98 cycles.

### SNP-array genotyping

Genomic DNA was extracted from 2 to 3 million PBMCs using QIAamp® DNA blood Mini Kit according to the manufacturer’s protocol, and samples were genotyped using the Illumina Infinium® HTS Assay. The intensity data files collected by the Illumina HiScan system were analyzed with the Illumina GenomeStudio software, and PLINK binary genotyping data were extracted for downstream analysis.

### Multi-parametric FACS symphony

An antibody staining panel consisting of 23 markers was designed for immunophenotyping of PBMCs populations using a variety of fluorochromes with minimum spectral cross-talk. This panel includes 16 lineage markers (e.g., CD45, CCR7, CD19, IgD, IgM, IgG, IgE, IgA, CD5, CD3, TCRab, TCRgd, CD4, CD8, CD14, and CD16) for detecting different populations of B cells (e.g., naïve IgD^+^/IgM^+^ B cells or switched IgG^+^/IgE^+^/IgA^+^ memory B cells), T cells (TCRab^+^ or TCRgd^+^ T cells), and monocytes (e.g., classical and non-classical monocytes). In addition, seven markers (e.g., CD20, HLA-DR, CD37, CD83, and LAPTM5, CD69, CD78) were selected based on the single-cell data for gene expression validations. The list of all antibodies used in this experiment as well as their corresponding clone, company, and catalogue number are listed in Additional file 2: Table S10. Frozen PBMCs were thawed as described, and aliquots of $2 \times 10^{6}$ cells were used for antibody staining. In order to optimize the concentration of each antibody in the panel, a single staining titration experiment with different concentration of each antibody (1 µl, 2 µl, and 4 µl) was performed using aliquots of $1 \times 10^{6}$ PBMCs derived from a healthy donor. For each antibody, the concentration at which the signal intensity gained the saturation point was selected as the optimal antibody concentration per million cells (Additional file 2: Table S10). Except for CCR7 antibody which required a pre-incubation step at 37 °C for 10 min, all other antibodies were incubated with cells for 30 min at 4 °C. In order to avoid any cross binding between TCR and CD3 antibodies, a sequential staining strategy was applied for these antibodies. After incubation with fluorescence-conjugated antibodies, the cells were washed and re-suspended in buffer solution (PBS containing 5% FBS and 2 mM EDTA). The stained cells were kept on ice before loading into the FACS symphony machine and data acquisition. The single stained files from the optimal antibody concentration were used to establish the compensation matrix. Finally, immunophenotyping was performed using the BD Symphony® machine for 10 T1DM and 9 healthy samples (independent validation cohort) and a minimum of 50,000 events were acquired per sample (Additional file 1: Fig. S4a).

### Processing and clustering of FACS symphony data

The compensated flow cytometry data were gated in the FlowJo (V 10.6) software using the standard gating strategy for selecting cell population, singlets, live, and CD45^+^ immune cells. The compensated channel values of the final population (i.e., live, single, CD45^+^ cells) from case and control samples were exported as CSV files. Next, these files were imported into R, and after down-sampling of each sample into 10,000 single cells, they were concatenated into a Seurat object (Seurat package V3) containing 190,000 cells. The Seurat package functionalities for dimensional reduction using the Uniform Manifold Approximation and Projection (UMAP) method and the unsupervised graph based clustering using the Louvain algorithm were utilized to calculate UMAP embeddings and perform unsupervised clustering based on 12 lineage makers including CD3, TCRab, TCRgd, CD19, IgD, IgM, IgG, IgE, IgA, CD14, CD16, and HLADR (Additional file 1: Fig. S4b-c).

### Processing of genotyping data

The binary PLINK genotyping files were exported from the Illumina GenomeStudio software and converted into the VCF format using the PLINK toolkit [39]. The vcftool program [40] was used to filter out insertions/deletions (indels), multiallelic SNPs, and sex chromosome variants. The hg19 human reference genome was used as a template in vcftools to correct the reference and alternative alleles labels in the VCF file and to phase all genotypes. The Beagle toolkit [41] was utilized to perform the genomic imputation according to the human 1000 genome reference. The imputed variants with low confidence (*R*^2^ $< 0.9$) were excluded, and the confidently imputed variants were concatenated into the genotyped variants. The final VCF file comprising genotyped variations (750k variants) and confidently imputed variants (3.2 million variants) was sorted and used for the demultiplexing of scRNA-seq data at sample level.

### Demultiplexing and quality control of scRNA-seq data

The overall steps for data processing, quality control, and reference-based clustering of PBMCs (*n *= 186,671) are represented in Additional file 1: Fig. S1. The Cell Ranger (V2.0) (with hg19 as reference genome) and demuxlet tool kits [42] were used to demultiplex paired-end sequencing FASTQ files at the cell and sample level, respectively. As expected, a total of 28,498 cells (average doublet rate of 15.2%) were detected as cross-sample doublets by the demuxlet tool and were removed from the dataset (Additional file 1: Fig. S1b, d). To check the fidelity of sample assignment by the Demuxlet, sum of average natural logarithm (*nlog*) expression of Y and X chromosome genes for cells of each sample were calculated and shown in a scatter plot, where each dot represents a sample colored based on the gender status obtained from the clinical data. As expected, the cells from female donors were clearly separated from cells from male donors as the former showed negligible (noise-floor) expression of Y chromosome genes (Additional file 1: Fig. S1c). Genes with low coverage (expressed in less than 0.1% of cells) and cells showing higher than 7% mitochondrial gene expression were filtered out. Read counts were normalized and log transformed using the SCTransform normalization method implemented in Seurat package (V3.1) [43]. The RCA2 package [44] was used to perform reference-based clustering of cells into major immune cell types and apply cluster-specific cell filtering based on the distribution of number of detected genes (NODGs) across immune cell types. Accordingly, cells with $750<$ NODG $< 2500$, $750<$ NODG < 2750, and $125<$ NODG $< 1500$ were kept within clusters of pDCs, HSCs, and platelets, respectively. For rest of RCA clusters, cells with $500<$ NODG $< 2500$ were included. A total of 19,508 cells were excluded as noisy cells (dead cells, debris, potential undetected doublets) (Additional file 1: Fig. S1a; Methods). After removing potential doublets and low quality compromised cells, the DoubletFinder package [45] was used to detect and exclude 9498 cells as within-sample doublets (average within-sample doublet rate of 6.86%), resulting in a final clean QC-passed dataset of 129,167 single cells used in the downstream re-clustering, cell type annotation, and cleaning.

### scRNA-seq clustering and cell type annotation

**Fig. 1:**
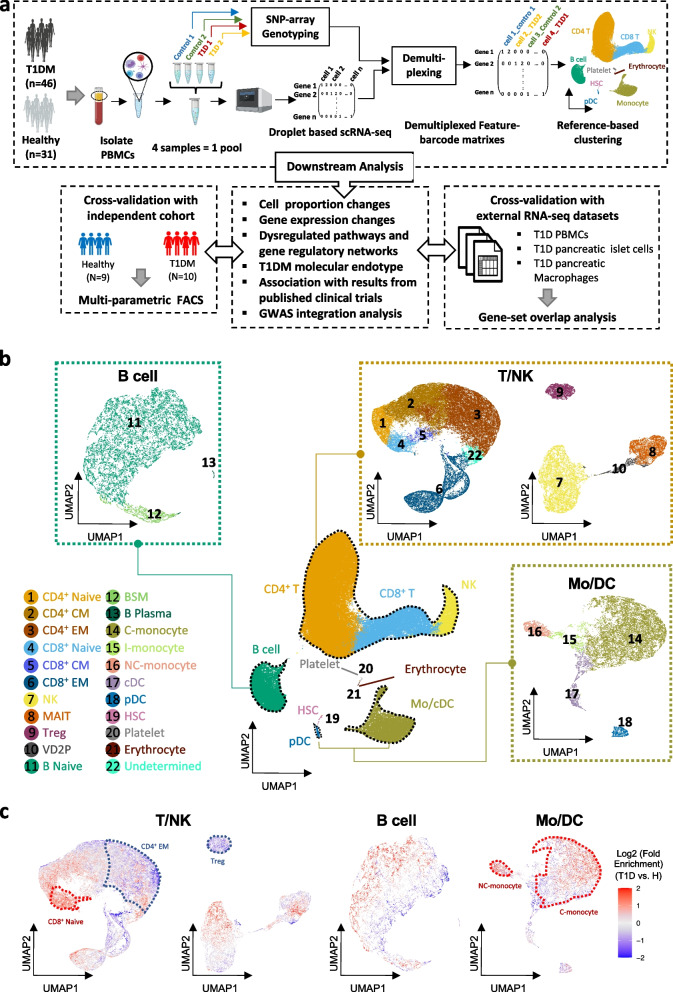
Multi-resolution clustering of PBMCs in T1DM. **a** Schematic representation of the study design for scRNA-seq and multi-parametric FACS profiling and data analysis of T1DM patients and healthy controls. **b** UMAP visualization of single cells in gene expression space. Central UMAP plot: low-resolution reference-based clustering to identify the three major immune compartments (T/NK, B, and Mo/DC). Each major cell type was then clustered individually at higher resolution, again using reference panels, to define 22 PBMC cell types. CM, central memory; EM, effector memory; Treg, regulatory T; MAIT, mucosal-associated invariant T; VD2P, gamma-delta T; NK, natural killer; BSM, B switched memory; C-monocyte, I-monocyte, and NC-monocyte: classical, intermediate, and non-classical monocyte; cDC, pDC, conventional, plasmacytoid dendritic cell; HSC, hematopoietic stem cell. **c** UMAP plot showing fold enrichment of T1DM vs healthy cells at each location in gene expression space. Red, blue: enriched, depleted in T1DM

Reference-based RCA clustering method supervised by a combination of different immune panels from RCA2 (V2) package [44] was used for clustering of QC-passed single-cell PBMCs. The details of reference panels used in this study are described as follows: (1) *Novershtern Panel*: including 966 feature genes across 15 PBMC cell types (i.e., CD4^+^ T naive, CD4^+^ TCM, CD4^+^ TEM, CD8^+^ T naive, CD8^+^ TCM, CD8^+^ TEM, NK, B-naive, B-SM, C-monocyte, NC-monocyte, cDCs, pDCs, platelets, HSCs) from the Novershtern et al. study [46]; (2) *Novershtern T Cell Panel*: including 420 feature genes across 6 T sub-populations (i.e., CD4^+^ T naive, CD4^+^ CM, CD4^+^ EM, CD8^+^ T naive, CD8^+^ CM, CD8^+^ EM) from the Novershtern et al. study [46]; (3) *Monaco TCell Panel*: including 551 feature genes across 15 T and NK sub-populations [i.e., CD4^+^ naive, CD8^+^ naive, CD8^+^ CM, CD8^+^ EM, CD8^+^ T effector (TE), MAIT, NK, T follicular helper (TFH), T helper 1 (Th1), Th17, Th1/Th17, Th2, T-reg, VD2 negative, VD2 positive] from the Monaco, G., et al. study [47]; (4) *Monaco B Cell Panel*: including 436 feature genes across 5 B sub-populations (i.e., B-naive, B non-switch memory (BNSM), BSM, plasmablasts, B exhausted (Bex)] from the Monaco, G., et al. study [47]; (5) *Monaco Myeloid Panel*: including 775 feature genes across 5 myeloid sub-populations (i.e., C-monocyte, I-monocyte, NC-monocyte, pDC, cDC) from the Monaco, G., et al. study [47]. To perform reference-based clustering supervised by the reference panels, the *dataProject* function from the RCA2 package was used to project log transformed corrected UMI count of each single cell into each cell type in the immune reference panels by calculating the Pearson correlation coefficient between the *nlog* transformed (SCTransformed normalized corrected UMI counts) vector from scRNA-seq and *log*10 expression vector from the reference bulk transcriptome across feature genes of the reference panel. This function generates the correlation coefficient matrix where each row is a cell type in the panel and each column is a single cell. After raising to the fourth power and row-wised z-transformation, the correlation matrix was used to calculate the correlation-based distance matrix. Next, the *dataClust* function from RCA2 (V2) package, which uses the internal *dataClust* function from *fastcluster* package, was used to apply the correlation-based distance matrix into average-linkage hierarchical clustering. It also takes advantage of the *cutreeDynamic* function from the WGCNA package to define cell clusters within the resulting dendrogram. To visualize the correlation-based distance matrix in a two-dimensional space, the UMAP dimensional reduction technique [48] was applied to the correlation-based distance matrix using the *computeUMAP* function from the RCA2 package. RCA clusters were annotated based on their correlation with immune cell types in each immune panels and expression of the canonical markers. Ambiguous RCA clusters with no correlation or strong correlation with more than one cell type in the panel were excluded from the dataset. First, in order to identify the major immune cell types, the QC-passed cells were projected into the *Novershtern Panel* resulting in 9 RCA clusters annotated as CD4^+^ T, CD8^+^ T, NK, B cell, monocyte, pDCs, platelets, HSCs, and erythrocytes (Fig. 1b, middle panel; Additional file 1: Fig. S2a). T/NK cells were further clustered into 11 T cell sub-populations by sequential projection into two T cell specific panels. First, T/NK cells were projected into the *Monaco T Cell Panel* where the cells with strong correlation with Treg, MAIT, gamma-delta T (VD2P), and NK cells were annotated accordingly and the remaining cells were projected into the *Novershtern T Cell Panel* resulting in six T sub-populations annotated as CD4^+^ T naive, CD4^+^ CM, CD4^+^ EM, CD8^+^ naive, CD8^+^ CM, CD8^+^ EM (Fig. 1b, upper-right panel; Additional file 1: Fig. S2c). B cells were projected into the *Monaco B Cell Panel* to identify and annotate three B sub-populations including B naive, BSM, and B plasma accordingly (Fig. 1b, upper left panel; Additional file 1: Fig. S2d). Likewise, projection of the monocyte/pDC cells into the *Monaco Myeloid Panel* revealed five myeloid sub-populations including C-monocyte, I-monocyte, NC-monocyte, cDC, and pDC (Fig. 1b, lower right panel; Additional file 1: Fig. S2e).

### Cell composition enrichment analysis at gene expression space

To evaluate the enrichment of cells from T1DM patients in gene expression space across immune cell types, the nearest 50 neighbor cells around each single cell in the gene expression principal component analysis (PCA) space were collected, and the *log*2 (fold-change ratio) of cells from T1DM samples relative to the cells from healthy subjects within nearest 50 neighbor cells was measured. These values were visualized for each single cell at the UMAP space.

### Identifying marker genes of PBMCs’ cell types

To define the marker genes for each immune cell type compared with rest of the cells, the negative binomial generalized linear model implemented in *FindAllmarker* function from seurat package was used, while the sequencing library IDs (batch) and the disease status (healthy/T1DM) were added as variables to regress out. Only genes which were detected in at least 25% of the cells in any of the two testing groups were included in the model. Genes with average *nlog* (fold-change) higher than 0.25 and FDR *q*-value less than 0.05 were selected as the marker genes for each immune cell type.

### Differential cell composition analysis

To identify cell composition aberrations with taking into account the effect of independent variables, a multiple regression analysis based on the general additive model (GAM) was applied using the *gam* function from the mgcv R package [49]. This model benefits from properties of both generalized linear (GLM) models and additive models [50]. Various independent variables, including disease status (T1DM = 1, healthy = 0), sex (male = 1, female = 0), number of T1DM risk HLA haplotypes, and the GADA titer were included into the model to estimate their effect on predicting the relative proportion of immune cell types (% of PBMC). The regression coefficients were estimated by the model and the F test statistics was applied to compute *p*-values. By default, the relative proportion is considered as Gaussian response. In all analysis, the *p*-value less than 0.05 was considered statistically significant.

### Pseudo-bulk differential gene expression analysis

The DESeq2 (v.1.30.1) package was used to perform differential gene expression analysis on pseudo-bulk transcriptome profiles of T1DM and healthy samples. First, the pseudo-bulk expression matrices were calculated by summing UMI counts across cells as suggested by DESeq2. The shrunken log2 (fold-change) and standard error were measured by the *ashr* algorithm. Pseudo-bulk samples resulting from summing-up of less than 5 cells as well as genes that were detected in less than 5% of individuals were excluded from the analysis. Finally, genes with an absolute *log*2 (fold-change) $\ge log2(1.3)$ and FDR *q-*value (Benjamini-Hochberg) $\le 0.05$ were defined as DEGs.

### Pathway enrichment analysis of T1DM-associated DEGs

Both over-representation and gene set enrichment analysis (GSEA) were performed using the ClusterProfiler R package [51]. The *enrichGO* function was used to perform the over-representation analysis on the list of DEGs from each k-mean cluster with the following settings: OrgDb = org.Hs.eg.db, ont = “BP,” pAdjustMethod = “BH,” pvalueCutoff = 0.05, qvalueCutoff = 0.05, minGSSize = 10, maxGSSize = 500. To perform GSEA analysis for each cell type, the *gseGO* function was applied on the list of DEGs ranked by the differential expression score (DES) [$|log2 (Fold-Change)\vert \times -log10 (FDR \textit{q-value})$] using the following settings: OrgDb = org.Hs.eg.db, ont = “BP,” keyType = “ENTREZID,” pAdjustMethod = “BH,” pvalueCutoff = 0.05, exponent = 0, nPerm = 10, 000, minGSSize = 15, and maxGSSize = 500. The *simplify* function was used to simplify the enriched terms. The top 3 significantly enriched terms (ranked by NES) and the cell type-specific terms of each cell type were selected for visualization.

### Single-cell regulon activity inference and differential regulon activity (DAR) analysis

The well-established Single-Cell rEgulatory Network Inference and Clustering (SCENIC) algorithm [52] was utilized to infer the regulon activity in single-cell PBMCs. The SCENIC pipeline with default settings was applied on the whole scRNAseq PBMC dataset and the final matrix of 260 inferred regulons (rows) with their corresponding regulon activity score (RAS) across 117,737 single cells (columns) was extracted as a Seurat object for the downstream analysis. Next, the unpaired Wilcoxon test, implemented in the Seurat *FindAllmarker* function, was used to identify the list of marker regulons within each immune cell type compared with the rest of the immune cells. For each cell type, the significant upregulated regulons [nlog (fold-change) > 0 and FDR *q-value *$< 0.05$)] were ranked based on their FDR *q*-values, and the top 5 regulons were selected as the marker regulons of that cell type. The same approach was used to identify the DARs between T1DM and healthy samples across immune cell types. Accordingly, within each cell type, all significant upregulated [nlog (fold-change) > 0] and downregulated [nlog (fold-change) $< 0$] regulons with FDR *q-*value $\le 0.05$ were selected as DARs in that cell type and were ranked based on their FDR *q*-value.

### Gene overlap analysis

The *GeneOverlap* R package [53] was used to compare the level and significance of overlap between upregulated and downregulated DEGs of T/NK cells, B cells, and monocytes from the current study with reported DEGs from previous RNA-seq profiling studies in T1DM. To do so, first, the list of up and down regulated DEGs was fetched from three studies including (1) longitudinal bulk RNA-seq study of FACS-sorted T cells and PBMCs from seven genetically at-risk case-control pairs at 3, 6, 12, 18, 24, and 36 months of age [24]; (2) bulk RNA-seq of human pancreatic $\beta$ cells isolated from 4 T1DM and 12 healthy donors [18]; and (3) scRNA-seq of macrophages within human cultured islets isolated from 5 T1DM and 11 healthy donors [29]. The *newGOM* function from the *GeneOverlap* package was used to apply the Fisher exact test and calculate the statistical FDR *q*-value (Benjamini-Hochberg) of intersections.

### TMZ score analysis

For each cell type, sample-level pseudo-bulk expression of DEGs were *z*-transformed across samples, and TMZ score was calculated by the following equation:

$$\begin{aligned} TMZ_i = \sum \limits _{j \in U_i}\frac{z_{ij}}{\Vert U_i\Vert } - \sum \limits _{k \in D_i}\frac{z_{ik}}{\Vert D_i\Vert } \end{aligned}$$

where *i* is the cell type *i*. The $z_{ij}$ and $z_{ik}$ represent the expression *z*-score for genes *j* and *k* in cell type *i*, respectively. Finally, $U_i$ and $D_i$ indicate the set of upregulated and downregulated DEGs in cell type *i*, respectively. The norm notation denotes the size of the set (i.e., the number of upregulated or downregulated genes in that cell type). Lastly, the average TMZ score is simply the mean of TMZ score across all cell types.

### TMZ score validation method

After TMZ score is calculated as explained, to accommodate calculation of TMZ score for bulk RNA-seq from other dataset, DEGs coming from each cell types were grouped into 3 major cell types namely, T/NK, B, and monocytes and 3 TMZ scores were created. The set of upregulated and downregulated DEGs from each groups were used to measure the TMZ score for bulk RNA-seq data like in Fig. 5e and Additional file 1: Fig. S7c. Using the predetermined set of genes for each group, the TMZ scores are calculated on the bulk expression data. Subsequently, the retrieved TMZ scores are then quantile transformed per sample to scale the data to the same distribution as in the previously calculated TMZ scores in our single-cell dataset while preserving its gene expression ranking. This method of calculation allows for any new genetic information coming from only as few as 1 sample to have its own TMZ score referenced to our T1D single-cell expression data.

### T1DM GWAS enrichment analysis

T1DM GWAS summary statistics fetched from the most recent comprehensive study covering 61,947,369 genetic variants with 37,652,754 single-nucleotide polymorphism (SNPs) passed the general QC in 520,580 European samples (T1DM = 18,942; healthy = 501,638) [54]. T1D-associated DEGs were ranked based on differential expression score (DES) [$|log2(Fold-Change) \vert \times -log10 (FDR \textit{q-value})$]. Genes with *q-value* $< 0.1$ were filtered out and DES of remaining genes were normalized using min-max normalization method. Next, for each cell type, the ranked normalized DES values were inputted into CELLECT tool [55] to quantify their association with T1DM polygenetic GWAS signal (T1DM heritability) using MAGMA covariate analysis [56] with MAGMA window size = 100 kb. Thereby, CELLECT identified cell types whose DEGs were significantly correlated with T1DM heritability and genes that were in the top 200 MAGMA-inferred T1DM GWAS genes and top 30 percentile of normalized DES were set to be the definition of effector genes listed in Fig. 6b.

## Results

### Reference-based multi-resolution clustering of PBMCs reveals altered cell populations in T1DM

We leveraged the demuxlet protocol for pooled scRNA-seq [42] to profile gene expression in single PBMCs from stage 3 46 T1DM and 33 healthy samples matched for age, gender, and T1DM-associated HLA haplotype (Fig. 1a). After strict QC to discard low-quality cells and doublets (Methods), we obtained transcriptomic data on 117,737 PBMCs. All samples were collected from European donors at Ulm University Medical Centre [35](Methods). In brief, PBMC samples from fasting blood taken from well-controlled insulin-dependent subjects with autoimmune diabetes were used in this study. Disease duration was consistent across the majority of the cohort, ranging from 1 to 4 years in 70 percent of subjects. All T1DM cases were negative for C-peptide and were undergoing insulin treatment. Thus, none of the subjects met the criteria for the partial remission (see Methods for details of inclusion and exclusion criteria). Detailed clinical information including gender, age at blood collection, age at disease onset, GADA titer, and T1DM-associated HLA haplotypes is provided in Additional file 2: Table S1.

To minimize batch effects in jointly clustering and annotating cells, we applied a reference-based clustering method, Reference Component Analysis version 2 (RCA2, Methods) [44, 57]. First, using a reference panel of transcriptomes of major immune cell types [46], we identified and annotated nine high-level cell clusters that were well separated in gene expression space (Fig. 1b, middle panel, Additional file 1: Fig. S2a): CD4^+^ T, CD8^+^ T, NK, B, monocyte/conventional dendritic cells (Mo/cDC), plasmacytoid dendritic cells (pDCs), hematopoietic stem cells (HSCs), erythrocytes, and platelets. We excluded the three least abundant clusters (HSC, platelet and erythrocyte) and sought to identify sub-populations within the abundant cell types by first merging them into three immune compartments: T/NK, B, and Mo/DC (Mo/cDC plus pDC). Then, in a second, high-resolution round of cell type annotation, we clustered cells from each of the three compartments separately using additional reference panels (Methods), resulting in a total of 22 cell types, 21 of which could be annotated using canonical markers (Fig. 1b; Supp. Fig. Additional file 1: Fig. S2b-e, i; Methods). All clusters contained cells from all samples and pooled batches, suggesting that the clustering was mainly driven by biological differences between cell types rather than batch effects or sample specificity (Additional file 1: Fig. S2f, g).

Next, we investigated the enrichment of immune cells from T1DM patients relative to healthy controls at each location in gene expression space (Fig. 1c; Additional file 1: Fig. S3a, e, i; Methods). We observed depletion of Treg and CD4^+^ EM cells, as well as enrichment of CD8^+^ naive T cells, C-monocytes, and NC-monocytes in T1DM (Fig. 1c, Additional file 1: Fig. S2h). These results suggest that there could be systematic compositional changes in circulating immune cells in T1DM, particularly at the level of cell sub-populations.

### Cell composition changes of peripheral immune cells in T1DM

To systematically investigate cellular composition aberrations of peripheral immune cells in T1DM, we used linear multiple regression analysis with sex [58] and number of T1DM risk HLA haplotypes as confounders and cell type proportion (% of total PBMCs) as the dependent variable (Methods).

**Fig. 2:**
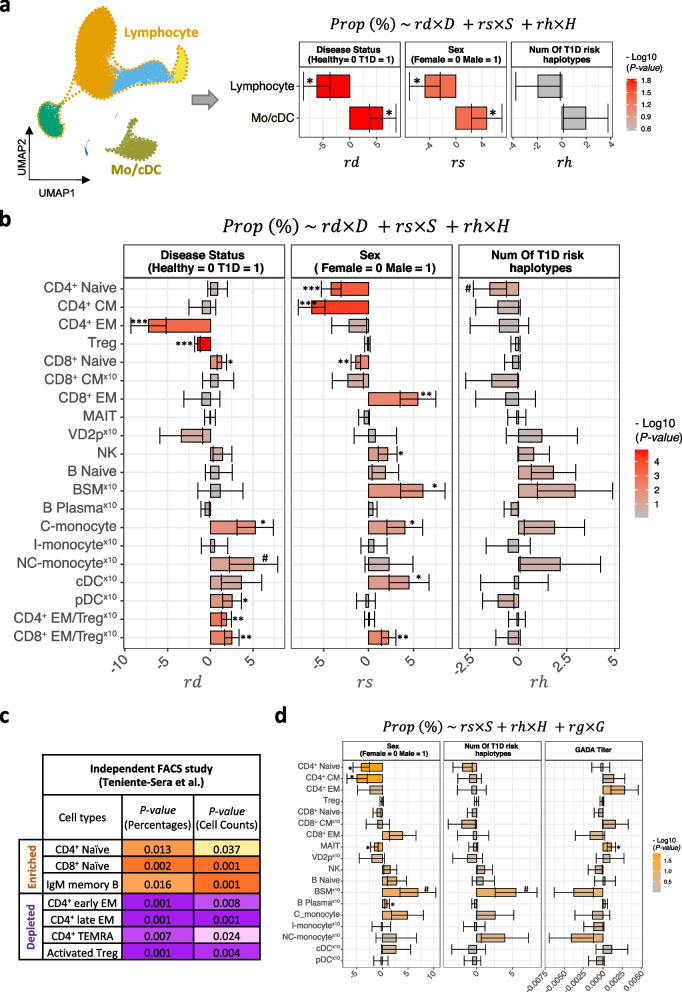
Cell composition alterations of peripheral immune cells in T1DM. **a** Bar plot of coefficients from multiple regression analysis of lymphocyte and Mo/cDC cell proportion (fraction of total PBMCs) against disease status, sex, and number of T1DM risk HLA haplotypes: 46 T1DM and 31 healthy samples. Error bars: standard error. **b** Same as **a**, for the 18 most abundant PBMC sub-populations, as well as the proportion ratios CD4^+^ EM/Treg and CD8^+^ EM/Treg. For easier visualization, the smaller values are multiplied by 10 (indicated with a superscript above the cell type label). **c** Cell composition changes from an independent multi-parametric FACS study of lymphoid cell types in T1DM [25]. Cell types that showed significant changes in both relative percentage and absolute cell count are shown. *P*-values from the original study: Mann-Whitney test. **d** Similar to **b**, on T1DM subjects alone, with GADA titer included as an additional independent variable. ^#^*p* $\le 0.09$, *p $\le 0.05$, **p $\le 0.01$, ***p $\le 0.001$

First, we examined compositional changes at the lowest level of resolution, by focusing on two broad compartments: lymphoid and Mo/cDC (Fig. 2a). Interestingly, the proportion of Mo/cDC cells increased significantly in T1DM, while the proportion of lymphoid cells decreased. Note that these two compartments together account for the vast majority of PBMCs, and thus one would expect that their relative proportions would shift in opposite directions. The Mo/cDC proportion was also significantly higher in male subjects, independent of T1DM status [58].

Next, we examined T1DM-associated composition changes at higher resolution, for the 18 most abundant PBMC sub-populations (Fig. 2b). Compared to controls, we noticed significant aberrations in the proportion of 5 immune cell types. CD4^+^ EM cells were strongly depleted in T1DM PBMCs, perhaps due to their recruitment to the para-pancreatic lymph nodes and pancreatic islets where they are shown to establish immunological synapses with antigen presenting cells (i.e., pDCs, B cells, and $\beta$ cells) and mediate the inflammatory responses against $\beta$ cells autoantigens [59–61]. Defects in immune suppressing function of Tregs have been reported in T1DM and other autoimmune disease [62–64]. Consistently, our scRNA-seq data revealed significant decrease in relative proportion of Tregs in PBMCs of T1DM patients (Fig. 2b), suggesting that, in addition to functional changes in Tregs, a reduction in their abundance could potentially contribute to the loss of peripheral tolerance in T1DM. Interestingly, in line with previous studies reporting higher ratio of naive/effector T cells in peripheral blood of T1DM patients [25, 61], we also identified enrichment of CD8^+^ naive T cells in PBMCs of T1DM patients (Fig. 2b). This observation supports the extensive literature showing a crucial role for CD8^+^ T cells in destructing of $\beta$cells during T1DM pathogenesis [61, 65–67]. Finally, we observed a higher proportion of C-monocytes and pDCs in peripheral blood of T1DM patients (Fig. 2b). We note that some of these T1DM-associated cell population changes may overlap those associated with aging [68].

We also examined the ratio of CD4^+^ and CD8^+^ EM cells to Treg cells, as a measure of T cell activation. Interestingly, both ratios were significantly elevated in T1DM (Fig. 2b). Consistent with previous studies [58], sex was again a significant confounding factor for multiple PBMC sub-populations. However, the number of HLA risk haplotypes had no significant effect.

Consistently, our multi-parametric FACS profiling of 190,000 cells from of 10 stage 3 T1DM and 9 healthy samples (10,000 cells per sample) (Additional file 1: Fig. S4a-c; Methods) validated the depletion of CD4^+^ T cells in T1DM (*p-*value = 0.037, Additional file 1: Fig. S4d-e). Likewise, it showed a trend towards higher proportions of CD8^+^ T and C-monocyte cells (Additional file 1: Fig. S4d-e). The cell composition shifts observed in our analysis were also supported by an independent multi-parametric FACS study [25] of B and T lymphocytes in T1DM. Consistently again, this study reported significant depletion of CD4^+^ early, late, and terminally differentiated EM (TEMRA) T cells as well as activated Tregs (Fig. 2c). Enrichment of CD8^+^ naive T cells in T1DM was also supported. These results from FACS profiling validate and support our conclusions from scRNA-seq that specific peripheral cell proportions are significantly altered in T1DM. Nevertheless, our scRNA-seq results encompass a far larger set of markers than can be analyzed using low-plex methods such as FACS.

We then examined the consistency of our results from single-cell analysis of peripheral immune cells with previously reported cell composition changes in relevant tissues, namely T1DM pancreas and para-pancreatic lymph nodes. In line with our own finding that Treg cells were depleted in T1DM peripheral blood, depletion of this cell type was reported as a key feature of para-pancreatic lymph nodes in stage 3 T1DM [63]. Furthermore, two independent imaging mass cytometry studies revealed an excess of macrophages in stage 3 T1DM pancreatic islets, which may relate to the excess of circulating C-monocytes and NC-monocytes observed in our study. In contrast to the depletion of CD4^+^ EM T cells in circulation, the overlapping CD4^+^ helper T cell population was enriched in T1DM islets, suggesting recruitment of these cells to pancreas in the disease state [69, 70].

We also investigated concordant signatures between PBMC cell composition changes in organ-specific (manifest T1DM, our study) and systemic autoimmune disease (established systemic lupus erythematosus (SLE)) [71]. In both T1DM and SLE, the ratio of lymphocytes to Mo/cDC cells was reduced relative to controls. Along the same lines, C-monocytes were enriched in both autoimmune diseases (Additional file 1: Fig. S5a). However, there were also notable differences. For example, CD4^+^ naive T cells were depleted in SLE, whereas we observed depletion of Treg and CD4^+^ EM T cells in T1DM. Similarly, we observed enrichment of CD8^+^ naive T cells and pDCs in T1DM, but no corresponding shifts were observed in SLE. Thus, the systemic shifts in cell composition we observed in the organ-specific autoimmune disease T1DM were similar in some respects to, but also substantially different from, those seen in SLE.

Next, we analyzed the association of cell composition in T1DM with sex and two clinical features: number of HLA risk haplotypes and GADA titer. We found a positive correlation between GADA titer and the proportion of MAIT (*p* = 0.026) and CD4 TEM cells (*p* = 0.086), suggesting a possible role for these cell types in GAD autoimmunity (Fig. 2d).

### Cell type-specific gene expression changes of peripheral immune cells in T1DM

**Fig. 3:**
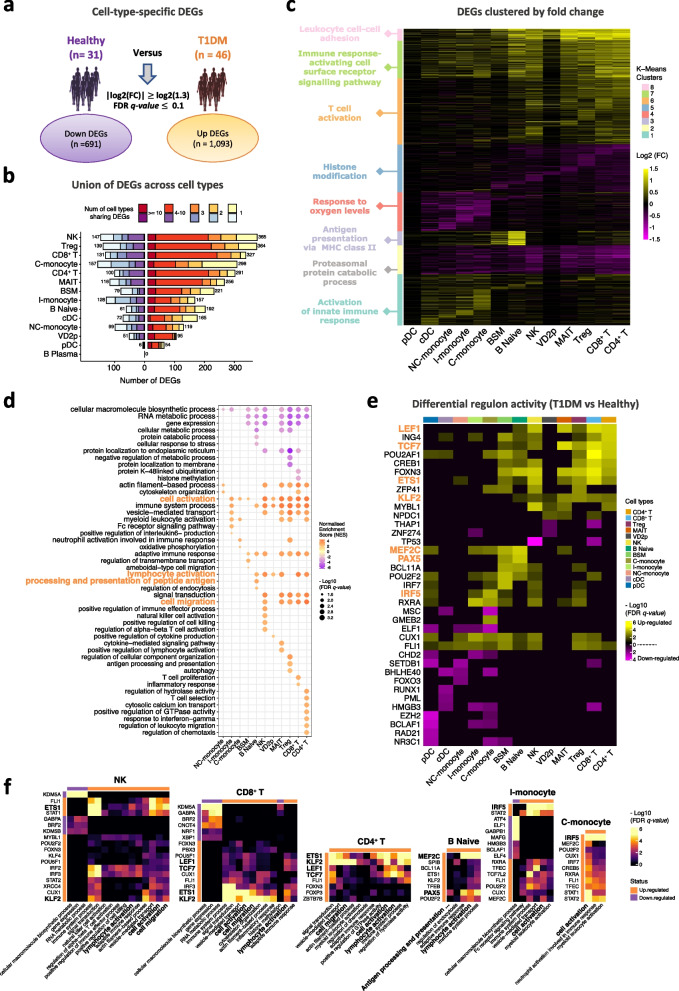
Cell type-specific gene expression changes in T1DM. **a** Total number of unique upregulated and downregulated DEGs in 14 peripheral immune cell types: 46 T1DM samples vs. 31 healthy. **b** Bar plot representing the number of DEGs within each cell type, with colored segments indicating the number of cell types that share the same DEG. **c** Heatmap of T1DM vs healthy *log*2 (fold-change) of 1784 DEGs ($691+1093$) across all immune cell types. Genes are clustered by their fold-change vectors using k-means. Each gene cluster is annotated by the most significantly enriched Gene Ontology (GO) term. **d** Gene set enrichment analysis (GSEA): dot plot of top shared and cell type-specific biological processes enriched in DEGs of each PBMC cell type, colored by normalized enrichment score (NES). Dot size: $-log10$ (FDR *q-*value). **e** Differential regulon activity: heatmap of transcription factor regulons with increased or decreased activity in T1DM (union of top 5 regulons in each of the 13 peripheral immune cell types). Color indicates $-log10$ (FDR *q-*value) of regulons upregulated (yellow) and downregulated (purple) in T1DM. **f** Overlap between GSEA leading-edge genes and significantly differential regulons in 6 peripheral cell types. Color indicates $-log10$ (FDR *q-*value) of overlap (Fisher’s exact test)

To identify transcriptome aberrations in peripheral immune cell types in T1DM, we performed pseudo-bulk differential gene expression analysis between the 46 cases and 31 control samples using DEseq2 [72] (Methods). Since we observed high overlap between DEGs of naive, CM, and EM T sub-populations within CD4^+^ T cells (data not shown), we merged these three sub-populations into a single CD4^+^ T cluster. The same approach was used to collapse cell sub-populations within CD8^+^ T cells. Remarkably, 1784 genes were identified as differentially expressed in at least one cell type ($|log2(Fold-Change) \vert \ge log2(1.3)$; FDR *q-*value $\le 0.1$), comprising 1093 upregulated and 691 downregulated DEGs (Fig. 3a; Additional file 1: Fig. S3b, f, j; Additional file 1: Fig. S6a; Additional file 2: Table S3). Our FACS panel for validating cell composition shifts included 8 of these DEGs, all of which showed protein expression changes in a direction consistent with transcriptome analysis, with 3/8 also showing statistical significance (Additional file 1: Fig. S4f). Interestingly, NK, Treg, CD8^+^ T cells comprised the top three cell types in terms of number of DEGs in T1DM (Fig. 3b), and $> 300$ genes were upregulated in each of these cell types. This indicates long-lasting systemic immune aberrations after type 1 disease manifestation, most prominently (though not exclusively) in cytotoxic cells and Treg cells that modulate their activity.

To identify gene modules among T1DM-associated DEGs, we clustered them by their cell type-specific *log*2 (fold-change) profile using k-means. This yielded 8 major DEG modules, most of which were preferentially altered in specific cell types. We annotated DEG clusters based on their most significantly enriched GO terms (Fig. 3c, Additional file 2: Table S4) (Methods). Notably, all three major cell lineages showed upregulation of the corresponding activation markers: “T cell activation” in T cells (cluster 6), “antigen presentation via MHC class II” in B cells (cluster 3), and “activation of innate immune responses” in myeloid cells (cluster 1). Consistently, gene set enrichment analyses (GSEA) of DEGs in each cell type (Methods) highlighted the enrichment of several overlapping biological terms including “cell activation” enriched in upregulated DEGs of most immune cell types and “processing and presentation of peptide antigens” enriched in up-DEGs of B cells. Furthermore, we observed significant association of T/NK up-DEGs with the term “cell migration” (Fig. 3d, Additional file 2: Table S5-S6). These results imply broad immune activation responses across PBMC cell lineages. However, the corresponding sets of DEGs were not entirely the same in T/NK, B, and monocyte populations (Additional file 2: Table S5-S6). These observations may suggest higher migratory potential of T/NK cells in T1DM, which may explain their active recruitment to the pancreatic islets and parapancreatic lymph nodes [63, 69, 70].

Next, we compared systemic transcriptomic aberrations in organ-specific (T1DM) and systemic (SLE) autoimmune diseases [71]. Interestingly, peripheral lymphoid and myeloid DEGs in T1DM significantly overlapped with the corresponding SLE DEGs (Additional file 1: Fig. S5b). These overlapping DEGs imply a shared loss of immune cell homeostasis in the two autoimmune diseases. For example, interferon gamma response genes were enriched in shared up-DEGs of T/NK cells, and myeloid cell activation genes were enriched in shared monocyte up-DEGs (Additional file 1: Fig. S5c, d, Additional file 2: Table S7). However, some functional perturbations appeared to be specific to T1DM up-DEGs, such as antigen processing and presentation capacity by B cells, driven by over-expression of a MHC class II cluster including *HLA-DM*, *HLA-DO*, *HLA-DRB1*, *HLA-DQB1*/*HLA-DQA1*, *CD74*, and *CTSS* [Fig. 3d; Additional file 1: Fig. S6b; [71]]. Thus, these results suggest significant similarities in the systemic immune response in T1DM and SLE, but also notable differences.

We then leveraged the SCENIC algorithm [52] (Methods) to identify transcription factors (TFs) that may contribute to gene and pathway dysregulation in T1DM. As a positive control, we first confirmed that the regulons (target genes) of canonical cell type marker TFs were upregulated in their corresponding cell types (Additional file 1: Fig. S6c; Methods). We then identified differentially active regulons (DARs) in T1DM versus healthy controls across the 13 immune cell types (Methods). We detected 123 significantly altered regulons (60 up, 63 down; FDR *q-*value $\le 0.05$) in at least one immune cell type (Additional file 1: Fig. S6d; Additional file 2: Table S9). Notably, we identified several instances of cell type-specific regulon activation in T1DM: *TCF7*, *LEF1*, *ETS1*, and *KLF2* in T/NK cells, *MEF2C* and *PCX5* in B cells, and *IRF5* in monocytes (Fig. 3e, orange; Additional file 1: Fig. S3c, g, k). To identify the TFs contributing to dysregulation of specific functional categories, we intersected the corresponding regulons with leading-edge DEGs of enriched GSEA terms in each immune cell type (Methods). This analysis revealed that distinct TFs may drive cell activation in each cell type: *TCF7*, *LEF1*, and *ETS1* in T/NK cells, *IRF5* in monocytes, and *PAX5* and *MEF2C* in B cells (Fig. 3f; Additional file 1: Fig. S3d, h, i). Furthermore, we identified *KLF2* as the master TF driving upregulation of cell migration genes in T/NK cells (Fig. 3f; Additional file 1: Fig. S3d) [73, 74]. Analysis of the signaling pathways upstream of these TFs that orchestrate T1DM-associated immune cell phenotypes may reveal new insights into the pathobiology of this autoimmune disease.

### Overlap with prognostic expression signatures and transcriptome changes in T1DM pancreas

**Fig. 4:**
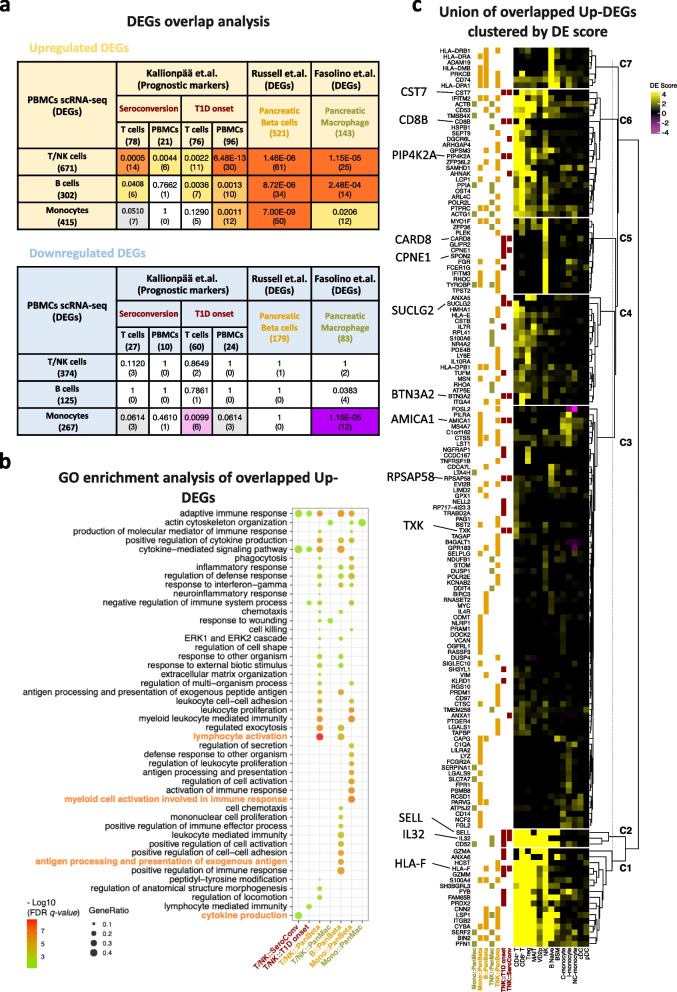
Overlap with prognostic expression signature and transcriptome changes in T1DM pancreas. **a** Overlap between T1DM versus healthy DEGs in PBMC cell types (this study) and prognostic markers and DEGs identified in other T1DM studies. Kallionpaa et al.: prognostic markers of seroconversion and T1DM onset. Russell et al., Fasolino et al.: T1DM versus healthy DEGs in primary pancreatic $\beta$ cells, cultured pancreatic macrophages. Statistical significance of overlap [Fisher’s exact test, $-log10$ (FDR *q-value*)] is indicated, with number of overlapping genes in parentheses. **b** Gene Ontology (GO) enrichment analysis: dot plot of biological processes enriched in overlapping up-DEGs in **a** colored by $-log10$ (FDR *q-value*). T/NK::SeroConv: T/NK DEGs that overlap prognostic markers of seroconversion in T cells or PBMCs (*n* = 20); T1DM onset: union of prognostic markers of T1DM onset in T cell and PBMCs; PanBeta: DEGs of pancreatic $\beta$ cells; PanMac: DEGs of pancreatic macrophages. Dot size: percentage of total DEGs in the given GO term. **c** Heatmap of differential expression (DE) scores [$|log2(Fold-Change) \vert \times -log10 (FDR \textit{q-value})$] for union of overlapping up-DEGs in **a**. DEGs are clustered by k-means into seven clusters (C1-7)

We hypothesized that some of the systemically altered immune cell genes in long-term T1DM could be perturbed even before disease onset. We therefore examined prognostic markers of seroconversion and T1DM onset previously identified in PBMCs of genetically at-risk infants [24] (Methods). Remarkably, these prognostic markers were significantly enriched for overlap with our T/NK up-DEGs (Fig. 4a, b). The overlapping genes were prominently associated with cytokine production and cytokine-mediated signaling (*TXK*, *HLA-F*, *ANXA1*, *IL32*, *FCER1G*, *IL7R*, *CARD8*, *CPNE1*, *BTN3A2*, and *CD8B*), suggesting that they may be involved in production and secretion of inflammatory cytokines by T/NK cells in the pre-disease state (Fig. 4c; Additional file 2: Table S8). In summary, numerous transcriptome aberrations of circulating immune cells in young adults with T1DM reflect continuation of pathogenic immune activation processes present at earlier stages of disease development.

Next, we asked whether the peripheral gene expression changes in T1DM were also reflected in the affected tissue, namely pancreatic islet cells [18, 29]. Indeed, T1DM versus healthy DEGs in pancreatic $\beta$ cells and pancreatic macrophages were highly enriched in peripheral up-DEGs of all three PBMC lineages (Fig. 4a, b). Consistently, the DEGs shared by pancreatic $\beta$ cells and PBMC were enriched for roles in lymphocyte (e.g., *ZFP36L2*, *HLA-E*, *ITGB2*, *LGALS1*, *TNFRSF1B*) and myeloid (e.g., *CD14*, *CTSC*, *LYZ*, *FGR*, *LGALS9*) cell activation and antigen presentation by B cells (e.g., *CD74* and several HLA genes) (Fig. 4c, orange; Additional file 2: Table S8). Importantly, 9 of the overlapping genes have genetic associations with T1DM risk: *GPSM3*, *HLA-DMB*, *HLA-DPA1*, *HLA-DPB1*, *HLA-DRA*, *HLA-DRB1*, *HLA-F*, *LST1*, *RPL41*. These results suggest significant similarity between dysregulated genes and pathways in circulatory immune cells and pancreatic islets in T1DM and support the relevance of systemic immune changes to the pathophysiology of T1DM pancreas.

### Transcriptome aberrations of peripheral immune cells define clinically relevant T1DM subtypes

**Fig. 5:**
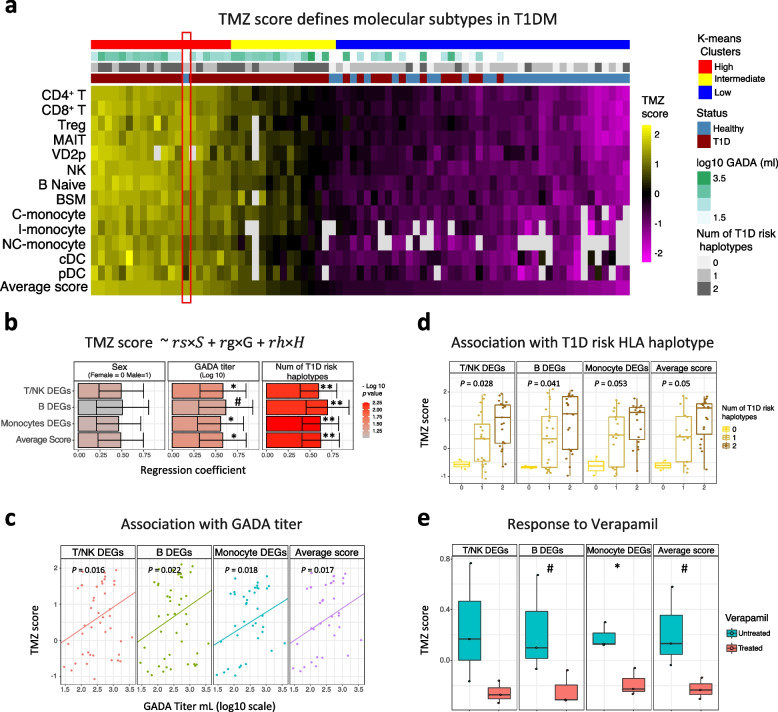
Transcriptome aberrations of peripheral immune cells define T1DM endotype. **a** TMZ score summarizing pseudo-bulk expression of T1DM-associated DEGs in each of 13 immune cell types: 46 T1DM and 31 Healthy. Samples were clustered by their TMZ score profiles using k-means. Missing values indicate insufficient cell count. **b** Bar plot of coefficients from multiple regression analysis of TMZ score against gender, GADA titer, and number of T1DM risk HLA haplotypes: 46 T1DM samples. Cell types avg: average of *z*-score across all cell types. Error bars: standard error. **c** Scatterplot showing the association of TMZ score with GADA titer across 46 T1DM samples. *p-*value: Spearman rank correlation. **d** Box plot representing TMZ score variation within each T1DM risk haplotype category. *p-*value: Kruskal-Wallis one-way ANOVA. **e** Effect of verapamil treatment [75] for 24 h versus untreated controls on TMZ score of human cultured islets. *p-*value: paired *t*-test. **b**, **e** ^#^*p* $\le 0.1$, *p $\le 0.05$, **p $\le 0.01$

We then asked if the expression of T1DM-associated DEGs could be used to define patient subtypes. First, in each cell type of each sample, we summarized the pseudo-bulk expression levels of the corresponding DEGs into a single TMZ score (Methods). As expected, this score largely segregated cases and controls. Remarkably, despite substantial differences between the 13 cell type-specific DEG sets, the corresponding 13 TMZ score were highly correlated (Pearson correlation coefficient $> 0.9$; Additional file 1: Fig. S7a). This result suggests that, in any individual sample, the 13 T1DM-associated molecular programs are activated to approximately the same extent (after *z*-score normalization). Thus, all 13 cell types are equally indicative of the strength of the systemic immune alteration in T1DM. We then clustered samples by their 13-dimensional TMZ score vectors into three groups to define molecular subtypes of T1DM representing high, intermediate, and low systemic immune response, respectively (Fig. 5a).

As expected, almost all healthy controls (29/31) belonged to the low-response T1DM subtype. Intriguingly, 13/46 T1DM cases were also assigned to the same subtype suggesting that a subset of diagnosed individuals may have relatively low systemic immune response, towards the upper end of healthy range (Fig. 5a). On the other hand, the intermediate- and high-responding subtypes were almost exclusively T1DM cases (33/35). Of note, the high-response group included one healthy control participant (Fig. 5a, red box). Remarkably, this participant was subsequently diagnosed with T1DM, 4 years after blood collection. Although anecdotal, this observation raises the possibility that systemic immune alterations may precede the development of insulin-dependence. Overall, these results suggest that T1DM associated DEGs of peripheral immune cells could be used to define molecular subtypes of T1DM.

Next, we asked if the observed variation in TMZ score across patients could be associated with two widely used clinical parameters: the number of T1DM risk haplotypes and GADA titer. Multiple linear regression analysis (Methods) revealed significant association of GADA titer as well as T1DM risk HLA haplotypes with TMZ score (Fig. 5b–d; Additional file 1: Fig. S7b). However, when we included age at diagnosis as an additional independent variable in the regression model for TMZ score, we did not find any significant association with this covariate (Additional file 1: Fig. S7b). This result could potentially be attributable to the fact that subjects with early onset T1DM (onset below 13 years) represented only a minority of our cohort (14/46, $30\%$).

Encouraged by the correspondence to clinical parameters, we asked if the TMZ score could be perturbed by treatment with immunomodulatory drugs in a non-antigen-specific manner. Specifically, we hypothesized that these drugs may reduce the TMZ score, i.e., they could move the immune profile of afflicted individuals closer to that of healthy controls. To test this hypothesis, we calculated TMZ score using bulk PBMCs transcriptome data from clinical trials for three drugs, namely teplizumab (anti-CD3) [76], abatacept (CTLA4 ig) [77], and rituximab (anti-CD20) [78] (Methods). In addition, we examined bulk transcriptome data from cultured pancreatic islets treated with verapamil [75, 79], relative to untreated controls. Verapamil, a drug that improved mixed-meal-stimulated C-peptide area under the curve and reduced the insulin requirement in a phase II trial [79], also reduced the TMZ score based on monocyte DEGs and had a marginally significant effect on the overall TMZ score (Fig. 5e; Additional file 1: Fig. S7c-e). While teplizumab had no detectable influence on the TMZ score, long-term treatment with abatacept, a drug designed to suppress T cell response, reduced the T/NK cell TMZ score. Rituximab, a drug targeting B cells, strongly reduced the B cell TMZ score on day 26, 4 days after the last dose was administered. These results suggest that the TMZ score could potentially be used to monitor drug response in pre-clinical and clinical T1DM studies and also as a readout in high-throughput screens.

### Cell type-specific DEGs enriched for T1DM genetic risk

**Fig. 6:**
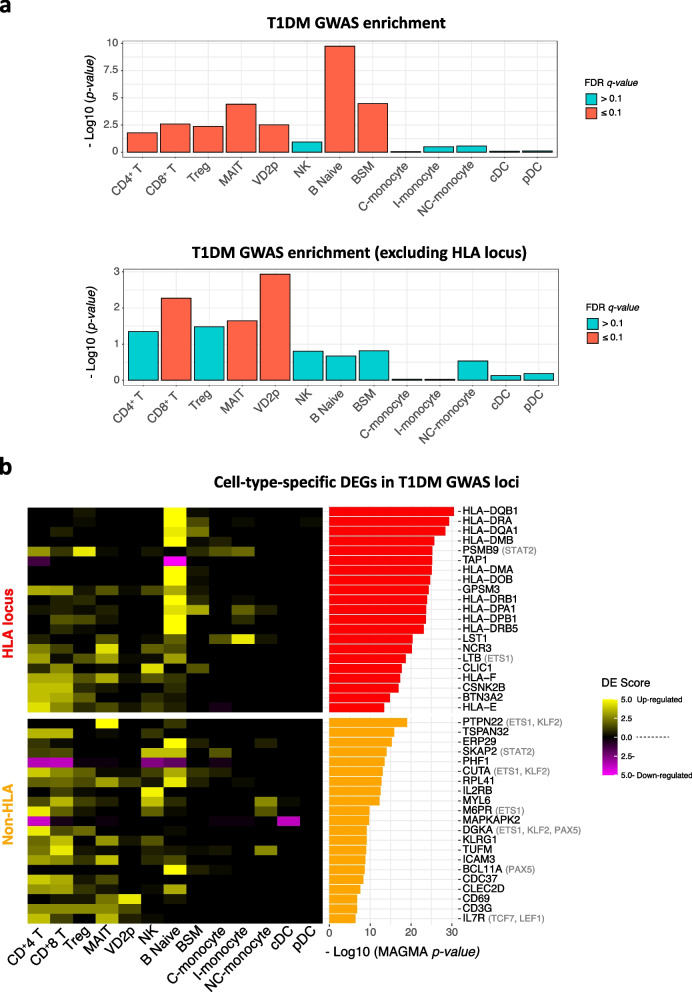
Cell type-specific DEGs enriched for T1DM genetic risk. **a** Significance of association between cell type-specific DEGs and T1DM risk loci. Upper panel: entire genome, lower panel: non-HLA loci. FDR *q-*values: Benjamini-Hochberg correction. **b** Heatmap of cell type-specific differential expression score (DES) of genes in T1DM GWAS loci. Gene within the upper 30th percentile of DES that also rank within top 200 in the genome by T1DM genetic risk score are shown. Genetic risk scores [$-log10$ (MAGMA *p-value*)] of individual genes are shown by the bar plot on the right. Black: DEGs, gray: upstream transcription factors inferred by regulon analysis

It is possible that some of the systemic immune DEGs detected in our single-cell analysis could be a consequence of chronic hyperglycemia, rather than linked to stage 3 T1DM. To prioritize cell types whose differential expression associates with disease causation, we used the CELLECT tool [55] to correlate the T1DM risk scores of genes (inferred from GWAS summary statistics [52]) with their cell type specific differential expression in T1DM versus healthy (Methods). This analysis highlighted DEGs in adaptive immune cell types as significantly enriched for T1DM genetic risk. DEGs in B naive cells showed the highest association with T1DM heritability, driven almost exclusively by multiple risk variants of strong effect in the HLA locus (Fig. 6a upper panel, b). This result indicates the crucial role of antigen presentation by B cells in T1DM pathogenesis [80].

To identify cell types associated with T1DM genetic risk in the rest of genome, we excluded the HLA locus and repeated the CELLECT analysis. In this case, DEGs in effector T cells (CD8^+^ T, MAIT, and VD2p) emerged as significantly enriched for T1DM heritability: *PTPN22*, *KLRG1*, *ICAM3*, *CLECC2D*, *CDC37*, *CD69*, *CD3G*, and *IL7R* (Fig. 6a lower panel, b). This result strengthens the evidence for the pathogenic role of effector T cells in T1DM [10, 60, 65–67, 81].

Also, we observed that 9 of the cell type-specific DEGs in T1DM GWAS loci belong to cell type-specific regulons driving upregulation of cell activation or cell migration genes displayed in Fig. 3f (Fig. 6b). These connections suggest that some of the genes influenced by T1DM-causing genetic variants may also be perturbed at the regulon level. In other words, these genes may represent convergence of cis- and trans-regulatory alterations at certain gene loci in T1DM.

Taken together, our GWAS integrative analysis highlights candidate cell types and corresponding DEGs that may mediate T1DM genetic risk in HLA and non-HLA loci. These genes could be targeted for functional assays in the corresponding cell types to investigate molecular mechanisms of T1DM pathogenesis.

## Discussion

We have constructed a cohort-scale single data set of peripheral immune cell states in T1DM as a resource to characterize systemic immune dysregulation in this prototypic endocrine organ-specific autoimmune disease. A key strength of our study is the use of single-cell transcriptome profiling of an autoimmune disease cohort, which provided sufficient resolution to detect cell type-specific molecular traits associated with T1DM. This approach also facilitated cross-validation of our results with T1DM markers found in diseased pancreatic tissue as well as circulating markers from single-cell analysis of SLE. Nevertheless, our study has some limitations. Firstly, this has been a cross-sectional study covering Stage 3 T1DM only. However, we note that the cross-sectional markers we identified from single-cell analysis showed significant overlap with previously detected prognostic markers. Secondly, we did not address signatures of auto-reactive T and B cells in T1DM.

In this study we detected a large number of DEGs between T1DM and control PBMCs (1784 DEGs across 13 cell types), indicating profound systemic immune aberrations in this organ-specific autoimmune disease (Fig. 3a, b). Remarkably, this was almost six-fold larger than the DEG set detected by scRNA-seq in the systemic autoimmune disease SLE (302 DEGs across 11 immune cell types, [71]). This increase could reflect an unexpectedly large systemic dimension to T1DM. We also identified substantial overlap in transcriptome changes between T1DM and SLE, including shared upregulation of cell activation genes in lymphoid and myeloid lineages [82–84]. This may further support the notion of a major systemic dimension to T1DM. These findings are in line with the observation that individuals diagnosed with T1DM are at greater risk of subsequently developing other autoimmune diseases such as celiac disease (small intestine) [85, 86], autoimmune gastritis [87, 88], autoimmune adrenalitis (Addison’s disease) [89], and quite common autoimmune thyroiditis (thyroid) [90, 91]. The negative clinical impact of the persistence of such immune cell changes is also evident as recurrent autoimmunity after pancreas or islet cell transplantation [92]. Immunosuppression is thus required to control immediate (re)activation of autoreactivity. Along the same lines, a large population-scale analysis showed that persistent immune activation and/or low-grade inflammation in T1DM appears to substantially increase the risk of cardiovascular disease in autoimmune diabetes subjects (hazard ratio:2.36), to a similar extent as SLE (hazard ratio:2.82) [93]. Our results, taken together with these epidemiological findings, make a case for expanding the scope of mechanistic studies of T1DM by examining systemic immunopathology and functional impairment in additional organs besides the pancreas.

Despite substantial overlaps between peripheral immune changes in T1DM and SLE, perturbation of antigen presentation by B cells (upregulation of MHC class II antigen/peptide presentation genes including *HLA-DR* and *-DQ*), was specific to T1DM (Fig. 3d; Additional file 1: Fig. S6b; [71]). In type 1 diabetes, it is well known that autoantigen presentation to CD4(+) T cells via MHC class II molecules plays a key role in the disease process [94–97]. This finding adds to the evidence from non-obese diabetic (NOD) mice [80, 98] supporting a key role for B cells as antigen presenting cells (APCs) in T1DM. Similarly, upregulation of cell migration genes in T/NK cells appears to be specific to T1DM (Fig. 3d; [71]). These results indicate qualitative differences between the systemic components of T1DM and SLE, and suggest that broader characterization of the peripheral immune aberrations of diverse autoimmune diseases could lead to new molecular assays for diagnosis and clinical monitoring. For instance, our T1DM-associated DEGs included a significant number of known prognostic markers of seroconversion and T1DM disease onset (Fig. 4a, c).

Remarkably, we observed significant overlap between the transcriptome aberrations of peripheral immune cells and those observed in pancreatic $\beta$ cells and macrophages in T1DM (Fig. 4a, c). This result suggests significant sharing of molecular mechanisms between peripheral immune cells and the relevant target tissue(s) [99]. Thus, our results provide further motivation for developing therapeutic strategies aimed at reversing the pathology of both pancreatic beta cells and circulating immune cells in T1DM [99–101].

Regulon analysis highlighted candidate master TFs driving cell type-specific changes in key functional programs in T1DM (Fig. 3e, f). Multiple lines of evidence support a role for these TFs in the pathophysiology of the corresponding cell types. For example, the WNT-pathway mediators *TCF7* and *LEF1*, which we identified as drivers of T/NK cell activation, are known to play a key role in maintenance of a Th17 stem-like population which can either give rise to effector Th17 cells or differentiate into highly tissue destructive Th17/Th1-like cells [10, 81, 102]. In addition, we identified *IRF5* as a potential driver of monocyte hyperactivation in T1DM. Consistently, genetic variants in this locus are associated with hyper-activation and functional aberrations of monocytes in T1DM and other systemic autoimmune disease such as SLE and Sjögren’s syndrome [103–106]. Lastly, our results indicate that *MEF2C*, a TF necessary for B cell survival and proliferation upon stimulation of antigen receptor [107] may also drive the abovementioned upregulation of antigen presentation genes in B cells. Targeting WNT [108, 109], interferon [110, 111], or other signaling pathways mediated by the identified master TFs could represent a promising avenue for therapeutics development in T1DM.

A key observation from our data is substantial heterogeneity across patients in the degree of T1DM-associated transcriptomic change (TMZ score). The clinical relevance of this heterogeneity is supported by the fact that the TMZ score showed significant correlation with GADA titer and number of HLA risk haplotypes, both of which are commonly used to infer disease risk [17, 19, 21] (Fig. 5). We therefore used the TMZ score to classify participants into high, intermediate, and low immune-responding molecular subtypes. As evidence for the relevance of this classification, the lone healthy subject assigned to the “high” subtype developed T1DM subsequently, 4 years after blood sampling. Lastly, analysis of transcriptomic response to drug treatment in vitro and in clinical trials revealed reductions in the TMZ score that were consistent with the mechanism of action and clinical response (Fig. 5e; Additional file 1: Fig. S7c-e). These results lay a foundation for further studies of the TMZ score as a readout in high-throughput screens, as a tool for T1DM patient stratification, and as an indicator of treatment response.

It should be noted that our dataset does not encompass the entire spectrum of autoimmune diabetes subtypes or endotypes (young-onset T1DM, adult-onset T1DM, as well as latent autoimmune diabetes of adults and other potential variants). Recognizing this limitation is crucial for a nuanced understanding of autoimmune diabetes and its various manifestations, emphasizing the need for future studies in a larger cohort, across a range of disease durations, to examine the temporal dynamics of this signature.

An additional constraint inherent in our study pertains to the absence of quantification of stimulated C-peptide levels in patients, thereby precluding the definitive exclusion of individuals identified as micro-secretors with retained C-peptide. We did, however, exclude T1D subjects with a “positive” fasting C-peptide level. We note that the sensitivity of the C-peptide assay does not compromise the substantive findings and primary conclusions derived from our investigation.

## Conclusions

In summary, our study provides a comprehensive characterization of systemic immune dysregulation in type 1 diabetes mellitus (T1DM) by constructing a large-scale single-cell dataset of peripheral immune cell states. Our findings reveal profound systemic immune aberrations in T1DM, suggesting an unexpectedly large systemic dimension to the disease, with a remarkable six-fold increase in the number of differentially expressed genes (DEGs) compared to systemic lupus erythematosus (SLE). The observed transcriptome changes in peripheral immune cells also exhibit significant overlap with those in pancreatic $\beta$ cells and macrophages, underscoring shared molecular mechanisms and offering insights for therapeutic strategies targeting both tissues. Moreover, our study introduces the T1DM-associated transcriptomic change (TMZ) score as a potential readout for high-throughput screens and an indicator of treatment response. We demonstrate substantial heterogeneity in T1DM-associated transcriptomic changes across patients, as reflected in the TMZ score, which correlates with disease risk indicators and provides a basis for patient stratification.

While our dataset significantly contributes to understanding systemic immunopathology in T1DM, we acknowledge its limitations, particularly the exclusion of the entire spectrum of autoimmune diabetes subtypes and the absence of stimulated C-peptide quantification. Future studies addressing these limitations in larger and more diverse cohorts will enhance our understanding of autoimmune diabetes dynamics over time.

### Supplementary information

**Additional file 1: Figure S1.** Computational workflow for data analysis and demuxlet quality control. **Figure S2.** RCA Clustering and annotation of 21 immune cell types. **Figure S3.** Illustration of T1DM and healthy cells, up/down-DEGs, and corresponding regulons/pathways in the UMAP representation of gene expression space. **Figure S4.** Multi-parametric FACS symphony validated the cellular and molecular aberrations in T1DM patients. **Figure S5.** Comparison of cellular and molecular changes in peripheral immune cells of SLE and T1DM versus healthy. **Figure S6.** Differential gene/regulon expression analysis in T1DM patients compared with healthy controls across 13 immune cell types. **Figure S7.** Association of TMZ score of peripheral immune cells with clinical features and response to immunotherapy in T1DM patients.

**Additional file 2: Table S1.** Clinical information of samples used for scRNA-seq. **Table S2.** Clinical information of samples used for FACS symphony. **Table S3.** Cell type-specific T1D DEGs. **Table S4.** Gene Ontology (GO) over-representaion results of T1D DEGs modules. **Table S5.** GSEA results of T1D DEGs across 13 immune cell types. **Table S6.** Filtered GSEA results (cell type-specific and top three GO terms in each cell types). **Table S7.** GO over-representaion results of T1D and SLE overlaping PBMC DEGs. **Table S8.** GO over-representaion results of overlaping T1D up-DEGs with prognostic expression signatures and transcriptome changes in T1D pancreas. **Table S9.** Cell type-specific T1D DARs. **Table S10.** List of antibodies used in this study.

## Data Availability

The raw scRNA-seq and snp-array genotyping data are available in the European Genome-phenome Archive (EGA) database and will be accessible upon reasonable request and approval of all authors (accession code EGAS50000000231) [https://ega-archive.org/studies/EGAS50000000231]. The processed single cell count expression matrix is publicly available through Synapse under the accession code syn53641849 [https://www.synapse.org/#!Synapse:syn53641849]. No custom code was used for any aspect of data processing or analysis in this study. All tools and packages used for data analysis in this study were published and are cited either in the main text or “Methods” section. Detailed data analysis procedures are described in the Methods section.

## Abbreviations

ANXA1: Annexin A1
APCs: Antigen presenting cells
BTN3A2: Butyrophilin subfamily 3 member A2
CARD8: Caspase recruitment domain family member 8
CD8B: CD8 subunit beta
CDC37: Cell division cycle 37
CD3G: CD3 gamma subunit
CLECC2D: C-type lectin domain family 2 member D
CPNE1: Copine 1
CTSC: Cathepsin C
CTSS: Cathepsin S
DEGs: Differentially expressed gene
DARs: Differentially active regulons
ETS1: ETS proto-oncogene 1
FCER1G: Fc epsilon receptor Ig
FDR: First-degree relatives
FGR: FGR proto-oncogene, Src family tyrosine kinase
GAD-65: Glutamic acid decarboxylase 65-kilodalton isoform
GADA: GAD autoantibody
GP: General population
GPSM3: G protein signaling modulator 3
GSEA: Gene set enrichment analysis
HLA: Human leukocyte antigen
IA-2A: Insulinoma-associated-2 autoantibodies
ICAM3: Intercellular adhesion molecule 3
IL32: Interleukin 32
IL7R: Interleukin 7 receptor
IRF5: Interferon regulatory factor 5
ITGB2: Integrin subunit beta 2
KLF2: Krüppel-like factor 2
KLRG1: Killer cell lectin like receptor G1
LEF1: Lymphoid enhancer-binding factor 1
LGALS1: Galectin 1
LGALS9: Galectin 9
LYZ: Lysozyme
MEF2C: Myocyte enhancer factor 2C
(NOD: Non-obese diabetic
PAX5: Paired box 5
PBMCs: Peripheral blood mononuclear cells
PTPN22: Protein tyrosine phosphatase non-receptor type 22
QC: Quality control
scRNA-seq:): Single-cell RNA sequencing
(SLE: Systemic lupus erythematosus
TCF7: Transcription factor 7
T1DM: Type 1 diabetes mellitus
TMZ: T1DM metagene *z*-score
TNFRSF1B: TNF receptor superfamily member 1B
TFs: Transcription factor
TXK: TXK tyrosine kinase
ZFP36L2: ZFP36 ring finger protein like 2
