# Supplementary material for: Systematic immune cell dysregulation and molecular subtypes revealed by single-cell RNA-seq of subjects with type 1 diabetes
Source: Genome Med. 2024 Mar 27;16:45. doi: 10.1186/s13073-024-01300-z (PMC10976681; doi:10.1186/s13073-024-01300-z)
Supplement: Supplementary file 1 — Additional file 1: Figure S1. Computational workflow for data analysis and demuxlet quality control. Figure S2. RCA Clustering and annotation of 21 immune cell types. Figure S3. Illustration of T1DM and healthy cells, up/down-DEGs, and corresponding regulons/pathways in the UMAP representation of gene expression space. Figure S4. Multi-parametric FACS symphony validated the cellular and molecular aberrations in T1DM patients. Figure S5. Comparison of cellular and molecular changes in peripheral immune cells of SLE and T1DM versus healthy. Figure S6. Differential gene/regulon expression analysis in T1DM patients compared with healthy controls across 13 immune cell types. Figure S7. Association of TMZ score of peripheral immune cells with clinical features and response to immunotherapy in T1DM patients. [file 13073_2024_1300_MOESM1_ESM.pdf]

Supplementary figures

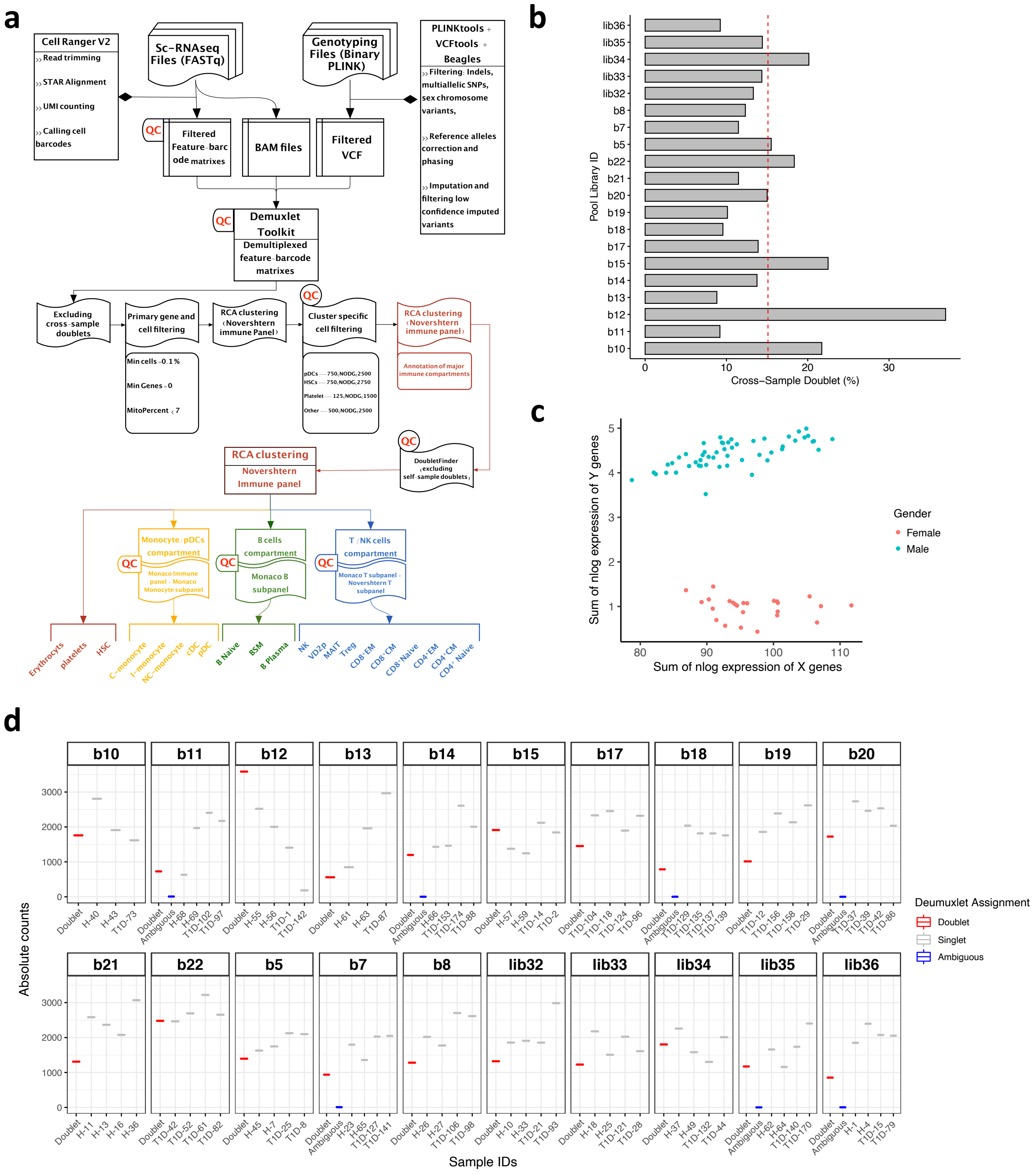

Fig. S 1. Computational workflow for data analysis and demuxlet quality control. a) Overall steps for data processing, quality control, and reference-based clustering of single cells data into 21 immune cell types are represented. QC: Quality control. **b)** Bar plot representing cross-sample doublet rates across 20 batches inferred by the demuxlet tool; The dashed red line represents the 15.2% average of the doublet rate across all batches. **c)** Scatter plot of sum of sample-level pseudo-bulk nlog expression of X and Y chromosome genes; Each dot is one sample coloured by the gender. **d)** Absolute cell counts of singlets (grey), doublets (red) and ambiguous (blue) barcodes assigned by the demuxlet tool across 20 batches.

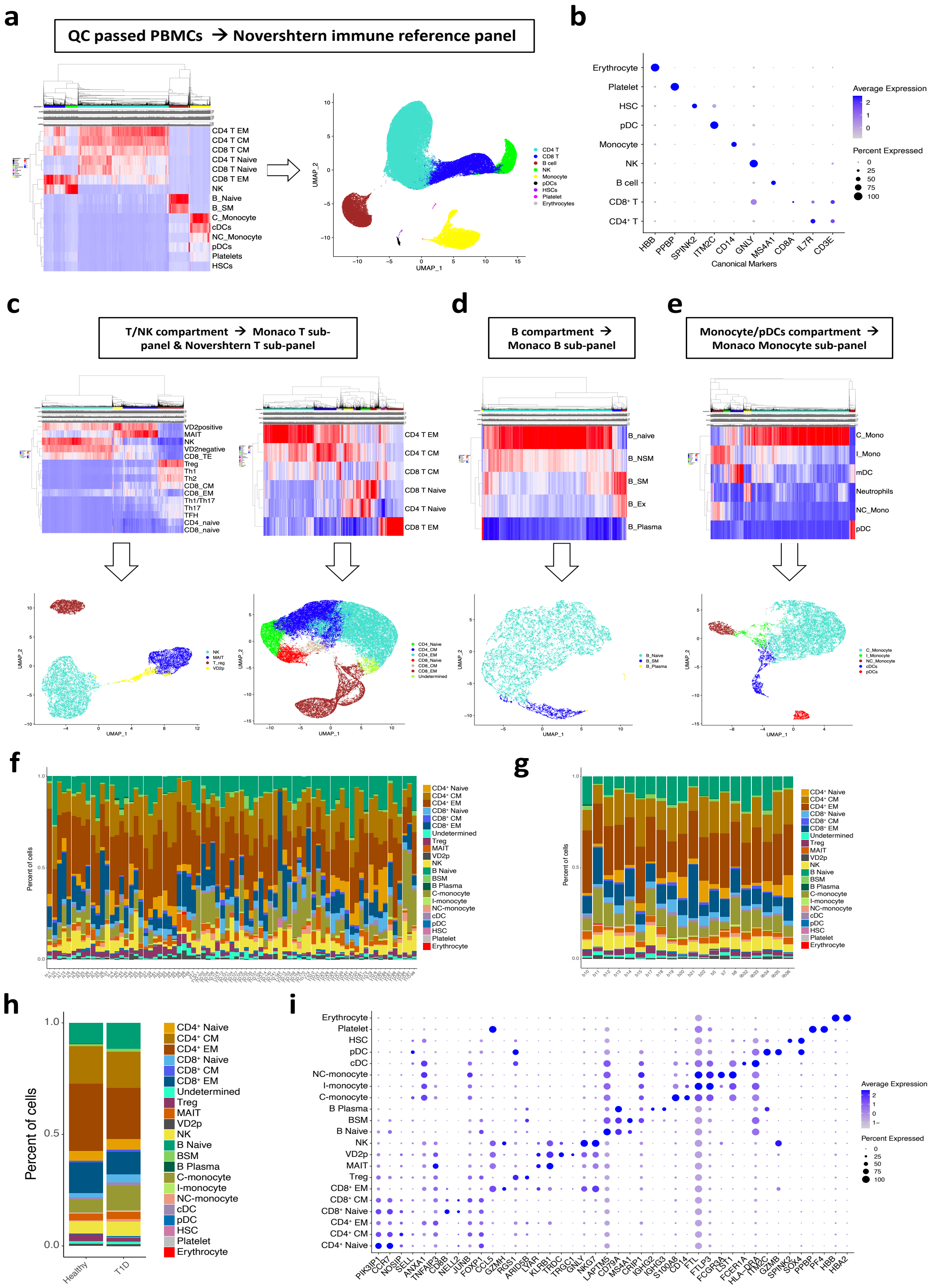

Fig. S 2. RCA Clustering and annotation of 21 immune cell types a) Left panel: projection heatmap representing the scaled pearson correlation coefficient between each single cell in the dataset and each immune cell type in the Novershtern immune reference panel across selected feature genes in the panel. Hierarchical clustering is applied to cluster the cells. Right panel: UMAP visualisation of the projection matrix colored by the RCA clusters. **b)** Average nlog expression of the canonical markers of major immune cell types is shown across the annotated RCA clusters. **c-e)** Projection heatmaps and their corresponding UMAP visualisation plots colored by the annotated RCA clusters are represented for T/NK (c), B (d), and Mo/DC (e) immune compartments after projection into their corresponding immune panels. **f-h)** Relative cell percentages of annotated RCA clusters across samples, batches, and total PBMCs of samples are shown in (f) and (g), and (h), respectively. **i)** Dot plot representing the scaled average nlog expression profile of the top two markers per cell type across final annotated RCA clusters.

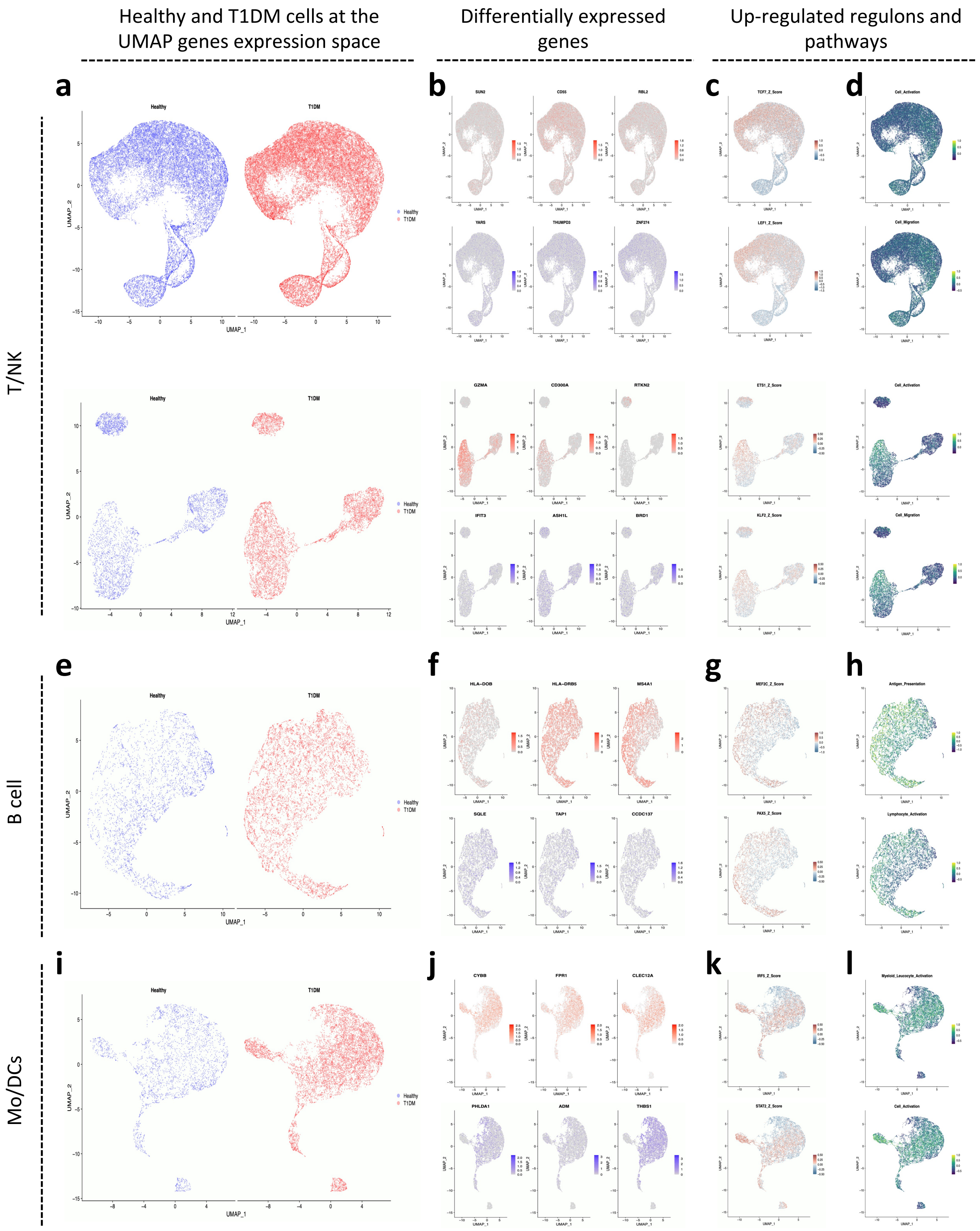

Fig. S 3. Illustration of T1DM and healthy cells, up/down-DEGs, and corresponding regulons/pathways in the UMAP representation of gene expression space. a-d) T/NK cells colored by a) healthy (blue) and T1DM (red) cells, b) nlog expression of top up- and down-DEGs (red and blue colors, respectively), c) metagene z-score (average across genes) of two up-regulated regulons, and d) metagene z-score of two biological pathways enriched in up-DEGs **e-h)** Similar plots, B cells **i-l)** Mo/Dcs.

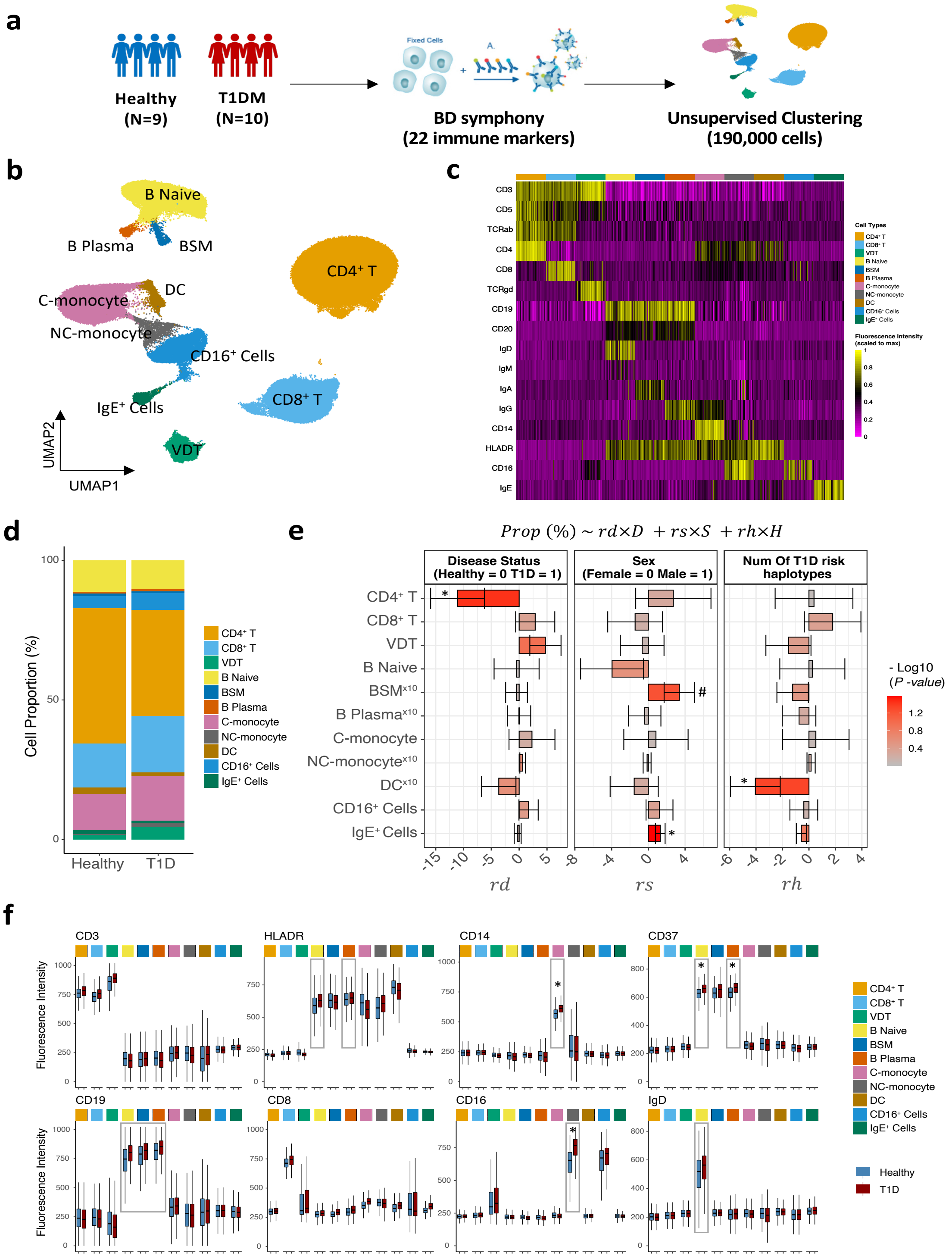

Fig. S 4. Multi-parametric FACS symphony validated the cellular and molecular aberrations in T1DM patients. **a)** Schematic representation of the study design for protein profiling of 22 immune markers in the validating cohort of 10 T1DM and 9 healthy controls using multi-parametric FACS symphony. **b)** UMAP visualisation of all cells (190,000 single alive CD45$^+$ cells captured by BD FACS symphony) coloured by Louvain clusters. Clusters are annotated based on expression of main lineage markers in the FACS panel. **c)** Heatmap plot representing scaled fluorescence intensity of main lineage markers in the FACS panel across all Louvain clusters. **d)** Relative cell percentages of annotated Louvain clusters across total PBMCs of samples. **e)** Bar plot of coefficients from multiple regression analysis of cell proportion of Louvain clusters (fraction of total PBMCs) against disease status, sex, and number of T1DM risk HLA haplotypes: 10 T1DM and 9 Healthy samples. Error bars: standard error. **f)** Comparing the protein expression (Unpaired Wilcoxon test) of selected genes in T1DM (red box plots) versus healthy controls (blue box plots) across annotated Louvain clusters. Protein markers corresponding to scRNA-seq DE genes in myeloid and B cells are indicated by gray rectangles #: $p \leq 0.1$, *: $p \leq 0.05$.

a

	PBMC scRNA-seq in SLE (Perez et al.)	
	Cell types	*P-value* (Percentages)
Enriched	C-monocyte	9.75E-7
Enriched	Cytotoxic CD8+ T	3.43E-4
Depleted	CD4+ T	5.58E-16
Depleted	CD4+ Naïve T	8.63E-21

b

DEGs overlap analysis

Up-regulated DEGs

PBMCs scRNA-seq (T1DM DEGs)	Perez et.al. (SLE DEGs)		
	T/NK cells (100)	B cells (39)	Monocyte (131)
T/NK cells (671)	1.92E-07 (23)	3.80E-05 (11)	0.0049 (17)
B cells (302)	2.00E-04 (11)	2.80E-05 (8)	0.0160 (9)
Monocytes (451)	2.04E-04 (13)	0.0070 (6)	5.56E-07 (20)

Down-regulated DEGs

PBMCs scRNA-seq (T1DM DEGs)	Perez et.al. (SLE DEGs)		
	T/NK cells (80)	B cells (27)	Monocyte (84)
T/NK cells (671)	0.30 (4)	0.70 (1)	0.20 (5)
B cells (302)	0.70 (1)	0.30 (1)	0.70 (1)
Monocytes (451)	2.32E-05 (11)	0.058 (3)	7.75E-06 (12)

c

Union of overlapped DEGs clustered by DE score

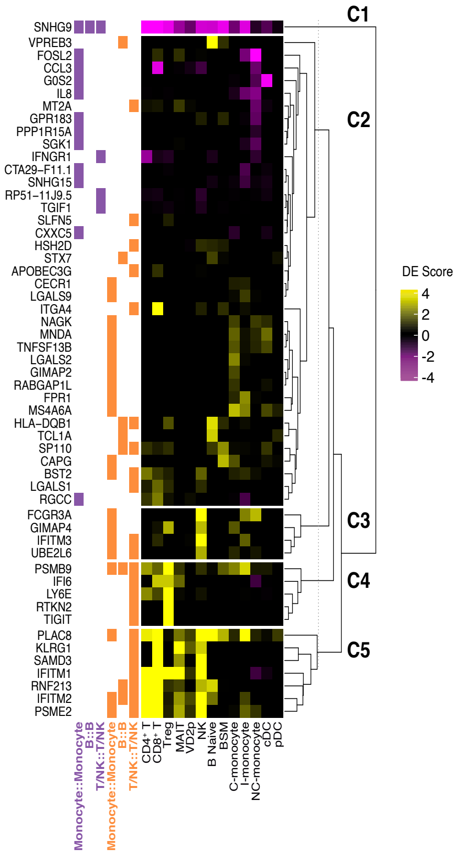

d

GO enrichment analysis of overlapped DEGs

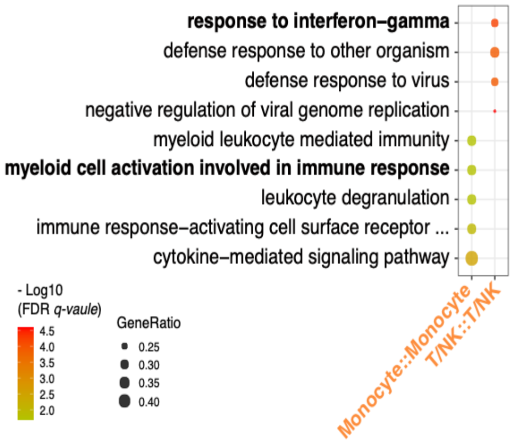

Fig. S 5. Comparison of cellular and molecular changes in peripheral immune cells of SLE and T1DM versus healthy a) Cell composition changes from the independent scRNA-seq study of PBMCs in SLE. Cell types that showed significant changes in their relative percentages compared with healthy controls are shown. P-value$_{\mathrm{meta:Fisher}}$ from the original study: weighted least squares (WLS). **b)** Overlap between T1DM versus healthy DEGs in PBMC cell types (this study) and DEGs identified in single cell SLE study. Statistical significance of overlap [Fisher's exact test, $-log10$(FDR q-$value$)] is indicated, with number of overlapping genes in parentheses. **c)** Heatmap of differential expression (DE) scores $[|log2(Fold-Change)| \times -log10(FDRq\text{-}value)]$ for union of overlapping DEGs in (b). DEGs are clustered by k-means into five clusters (C1-5) **d)** Gene Ontology (GO) over-representation analysis: dot plot of biological processes enriched in overlapping up-DEGs in (a) colored by $-log10$ (FDR q-$value$).Dot size: percentage of total DEGs in the given GO term.

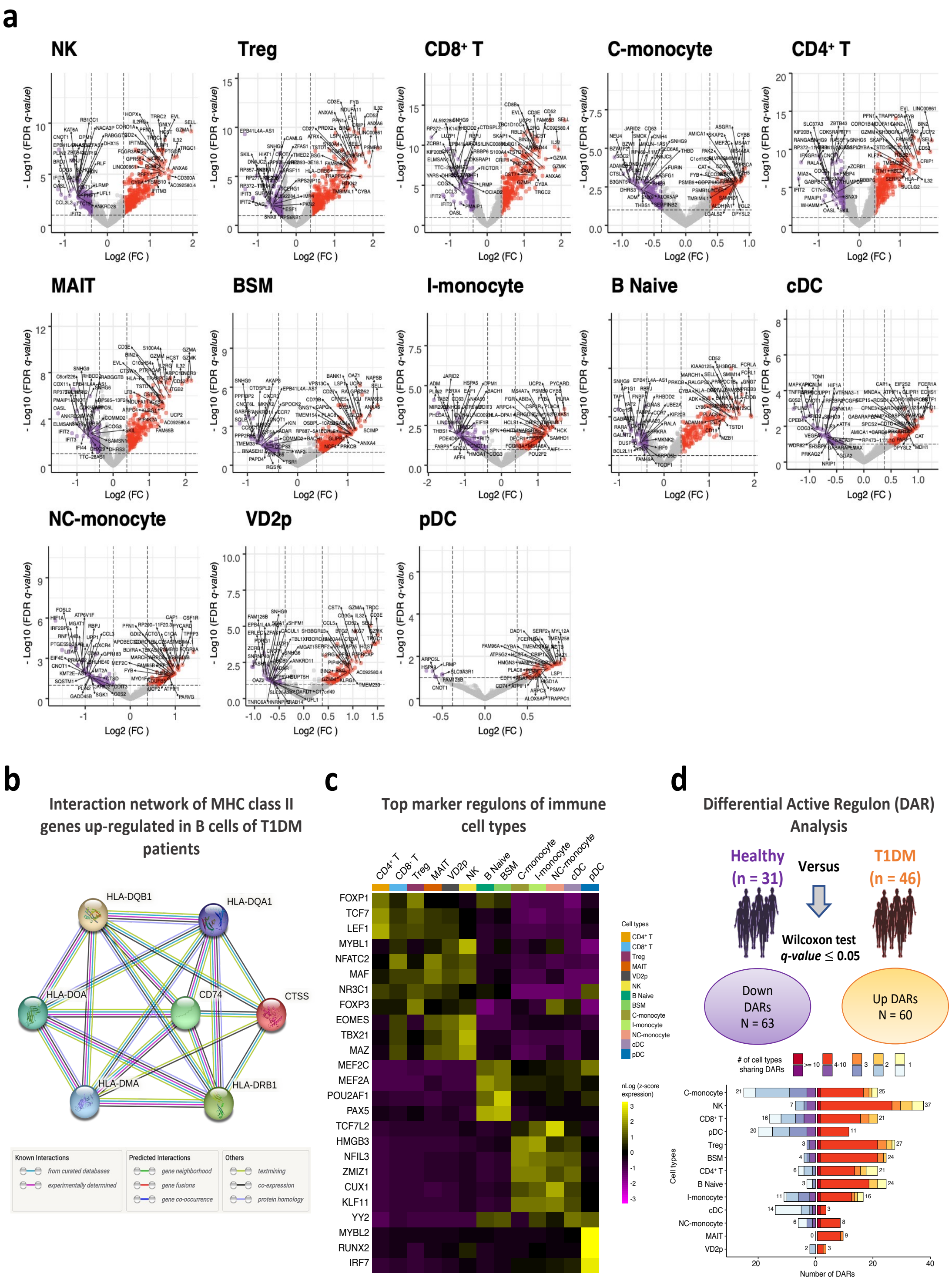

Fig. S 6. Differential gene/regulon expression analysis in T1DM patients compared with healthy controls across 13 immune cell types. a) Volcano plots of DEGs in T1DM patients across 13 immune cell types. Significant up- and down-regulated DEGs are colored in red and blue, respectively. Top 20 up- and down-regulated DEGs of each cell type [ranked by the *log*2 (fold change)] are labeled. **b)** Protein-protein interaction network of MHC class II genes up-regulated in B cells of T1DM patients (adopted from STRING database) **c)** Heatmap of scaled pseudo-bulk *nlog* Regulon Activity Score (RAS) of top three marker regulons across 13 immune cell types. **d)** Bar plot representing the number of up- and down-regulated differential active regulons (DARs) within each cell type, with colored segments indicating the number of cell types that share the same DARs.

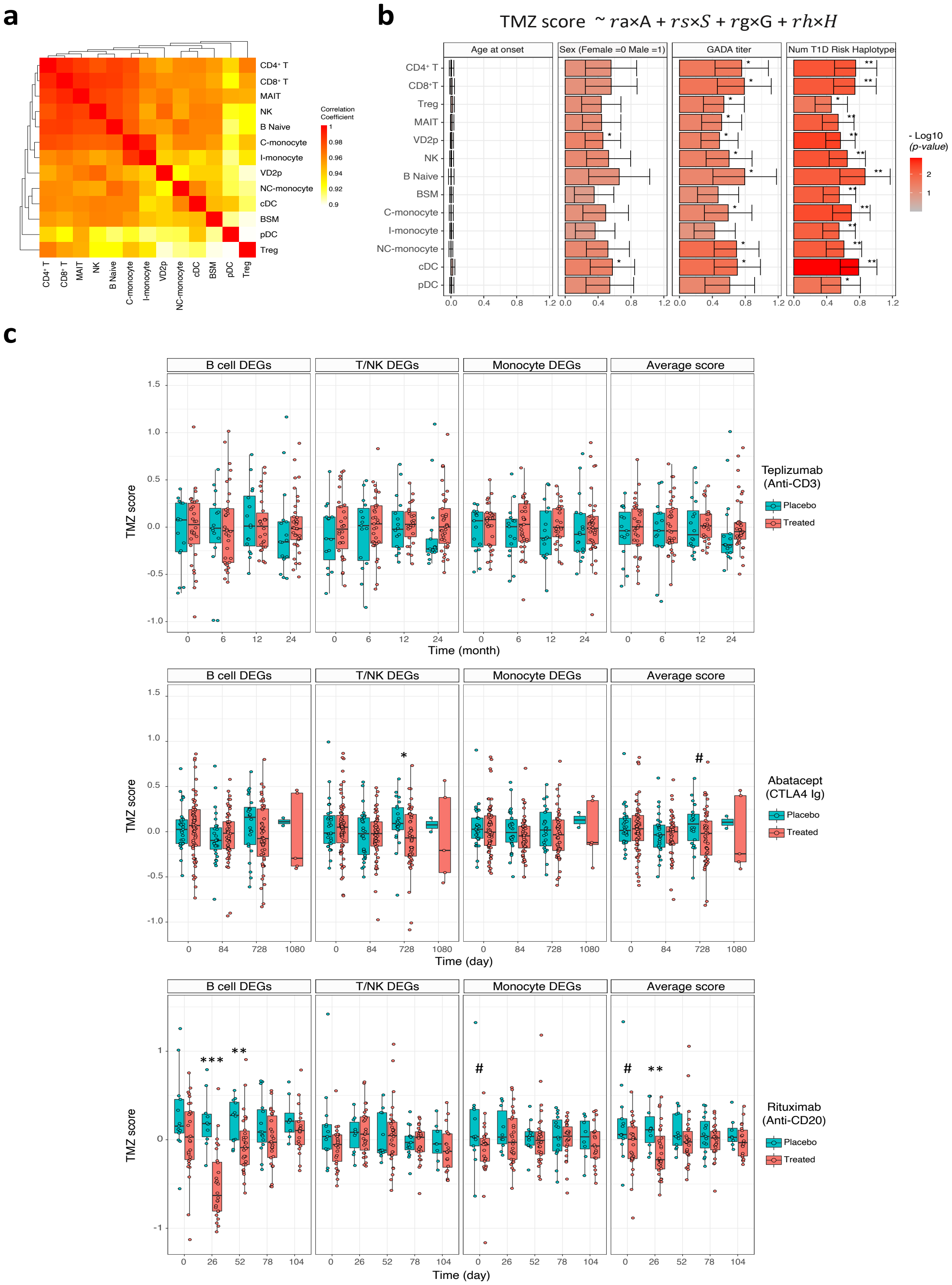

Fig. S 7. Association of TMZ score of peripheral immune cells with clinical features and response to immunotherapy in T1DM patients a) Heatmap plot showing the Pearson correlation coefficient of TMZ score of different immune cell types across samples with no missing cell type. **b)** Multiple regression analysis of TMZ score of 13 immune cell types in response to the age at diagnosis, sex, GADA titre, and number of T1DM risk HLA haplotypes: 46 T1DM cases. Bar plots represent the estimated regression coefficient of each variable in the regression model with the error bars representing the standard errors and the red colour gradient showing the -*log*10 (FDR *p-value*). **c-e)** Box plot representing the TMZ score of B , T/NK , monocytes or average of all cell types calculated from bulk-RNAseq data of PBMCs from T1DM samples receiving placebo or immunotherapies with teplizumab (c), abatacept (d), or rituximab (e) across different time points including before treatment (time point zero) and after treatment (other time points). Unpaired Mann-Whitney t-test was applied to estimate significant differences between placebo and active groups at each time point.#: $p \leq 0.1$, *: $p \leq 0.05$, **: $p \leq 0.01$, ***: $p \leq 0.001$.
